# Four New Isocoumarins and a New Natural Tryptamine with Antifungal Activities from a Mangrove Endophytic Fungus *Botryosphaeria ramosa* L29

**DOI:** 10.3390/md17020088

**Published:** 2019-02-01

**Authors:** Zhihui Wu, Jiaqing Chen, Xiaolin Zhang, Zelin Chen, Tong Li, Zhigang She, Weijia Ding, Chunyuan Li

**Affiliations:** 1 College of Materials and Energy, South China Agricultural University, Guangzhou 510642, China; w_zhi_hui@sina.com (Z.W.); ch_jiaqing@sina.com (J.C.); catherinezxll@sina.com (X.Z.); czrin@sina.cn (Z.C.); zjjlitong@sina.com (T.L.); 2 School of Chemistry, Sun Yat-Sen University, Guangzhou 510275, China; cesshzhg@mail.sysu.edu.cn

**Keywords:** isocoumarin, tryptamine, *Botryosphaeria ramose*, antifungal activity

## Abstract

Four new isocoumarin derivatives, botryospyrones A (**1**), B (**2**), C (**3**), and D (**4**), and a new natural tryptamine, (3a*S*, 8a*S*)-1-acetyl-1, 2, 3, 3a, 8, 8a-hexahydropyrrolo [2,3b] indol-3a-ol (**5**), were isolated from a marine mangrove endophytic fungus *Botryosphaeria ramosa* L29, obtained from the leaf of *Myoporum bontioides*. Their structures were elucidated using spectroscopic analysis. The absolute configurations of compounds **3**, **4**, and **5** were determined by comparison of their circular dichroism (CD) spectra with the calculated data. The inhibitory activities of compound **1** on *Fusarium oxysporum*, of compounds **2** and **3** on *F. oxysporum* and *Fusarium graminearum*, and of compound **5** on *F. oxysporum*, *Penicillium italicum*, and *F. graminearum* were higher than those of triadimefon, widely used as an agricultural fungicide. Compound **5** was produced after using the strategy we called “using inhibitory stress from components of the host” (UISCH), wherein (2*R*, 3*R*)-3, 5, 7-trihydroxyflavanone 3-acetate, a component of *M. bontioides* with weak growth inhibitory activity towards *B. ramosa* L29, was introduced into the culture medium.

## 1. Introduction

**Figure 1:**
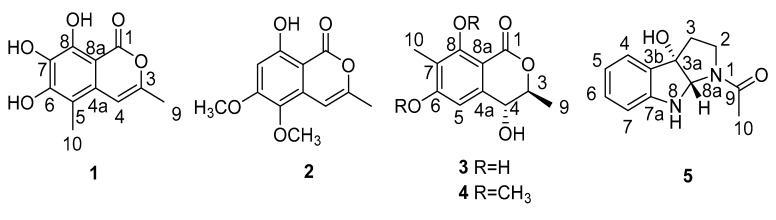
Chemical structures of the isolated compounds **1**–**5**.

Since the 1990s, marine-derived fungi including mangrove fungi have attracted considerable interest as a target source of bioactive natural products with chemodiversity [1,2,3]. *Myoporum bontioides* (Siebold and Zucc.) A. Gray (Scrophulariaceae) is one of the mangrove plants distributed along the coasts of Asian countries [4]. Our chemical investigations into this species have led to the discovery of various flavonoids, sesquiterpenoids, and phenylethanoids, among others [5]. Through our ongoing search for novel bioactive metabolites from the endophytic fungi of this plant [6,7,8,9], we have investigated the secondary metabolites of the fungus *Botryosphaeria ramosa* L29, the ethanolic extract of which was found to possess antifungal activity. Four new isocoumarin derivatives, named botryospyrones A (**1**), B (**2**), C (**3**), and D (**4**) (Figure 1) were isolated. However, under such a common cultural condition, the fungal strain faced no living stress from the environment. A plausible question is whether inhibitory stressors from components of the host under natural conditions might change the metabolites of the fungal strain. To provide an answer to this question, a strategy which we called “using inhibitory stress from components of the host” (UISCH) was used and prompted the endophyte to produce the novel natural product (3a*S*, 8a*S*)-1-acetyl-1, 2, 3, 3a, 8, 8a-hexahydropyrrolo [2,3b] indol-3a-ol (**5**) belonging to the tryptamine class. 

Herein, we report the details of the isolation, structure elucidation, and antifungal activity of these compounds. 

## 2. Results and Discussion

Compounds **1**–**4** were obtained from the fungus *Botryosphaeria ramosa* L29 grown in common autoclaved rice medium. Compound **5** was produced when using the UISCH strategy. The flavonoid (2*R*, 3*R*)-3, 5, 7-trihydroxyflavanone 3-acetate, a component of *M. bontioides* [10] which had been found by us to have 18.56% growth inhibition rate (0.25 mM) to the mycelium of the fungus *Botryosphaeria ramosa* L29, was added to the autoclaved rice medium. Under these conditions, compound **5** was generated and purified.

Compound **1** was isolated as a yellow powder. The high resolution electron spray ionization mass spectroscopy (HRESIMS) spectrum of **1** showed an ion peak at m/z 223.0608 ([M + H]^+^, calcd. for C_11_H_11_O_5_ 223.0606), corresponding to the molecular formula C_11_H_10_O_5_. The infrared radiation (IR) bands of **1** at 3244, 1682, 1622, and 1615 cm^−1^ represented the hydroxyl, ester carbonyl, and aromatic ring groups, respectively. The ^13^C NMR (Table 1) and heteronuclear singular quantum correlation (HSQC) spectrum of **1** showed 11 carbon signals consisting of two methyl groups, one sp^2^ methine group, seven olefinic quaternary carbons, and one ester carbonyl carbon. The ^1^H NMR (Table 1) and HSQC spectra, along with the molecular formula of **1** indicated signals for one hydrogen-bond hydroxyl group (*δ*_H_ 11.18), two aromatic hydroxyl groups (*δ*_H_ 5.38 and 6.42), one olefinic proton (*δ*_H_ 6.38), and two methyl groups (*δ*_H_ 2.18 and 2.29). These characteristics suggested that compound **1** might be an isocoumarin [11] substituted with three hydroxyl and two methyl groups. 

**Figure 2:**
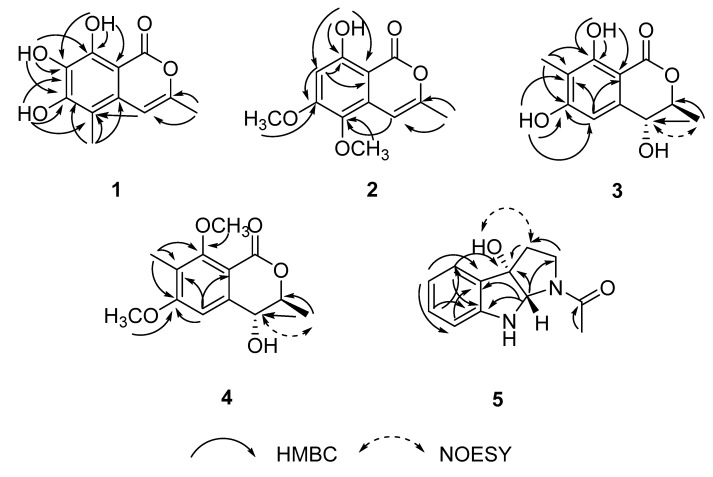
Key HMBC and NOESY correlations for compounds **1**–**5**.

The heteronuclear multiple bond correlation (HMBC) correlations (Figure 2) from 8-OH to C-7, C-8 and C-8a, from 7-OH to C-6, C-7, and C-8, from 6-OH to C-5, C-6 and C-7, and from H-10 to C-5, C-6 and C-4a indicated the positions of 6-OH, 7-OH, 8-OH, and CH_3_-10. In addition, the HMBC correlations from H-9 to C-3 and C-4, and from H-4 to C-5 suggested that CH_3_-9 was located at C-3. Therefore, the structure of compound **1** was determined as 6, 7, 8-trihydroxy-3, 5-dimethylisocoumarin, as shown in Figure 1.

Compound **2** was obtained as a colorless powder, with the molecular formula C_12_H_12_O_5_ determined by the HRESIMS at m/z 237.0765 ([M + H]^+^, calcd. C_12_H_13_O_5_ 237.0763). The NMR data (Table 1) and HSQC spectrum revealed that compound **2** shared the same isocoumarin skeleton as **1**. However, the CH_3_ at C-5 in **1** was replaced by a methoxy group in **2**, which was revealed by the HMBC correlations (Figure 2) from the proton of 5-OCH_3_ to C-5 and from H-4 to C-5. Moreover, the hydroxyl at C-7 in **1** was absent in **2**, and the hydroxyl at C-6 in **1** was changed to a methoxyl in **2**, which was supported by the HMBC correlations from H-7 to C-5, C-6, and C-8a, and from the proton of 6-OCH_3_ to C-6. Thus, compound **2** was elucidated as 8-hydroxy-5, 6-dimethoxy-3-methylisocoumarin (Figure 1).

**Figure 3:**
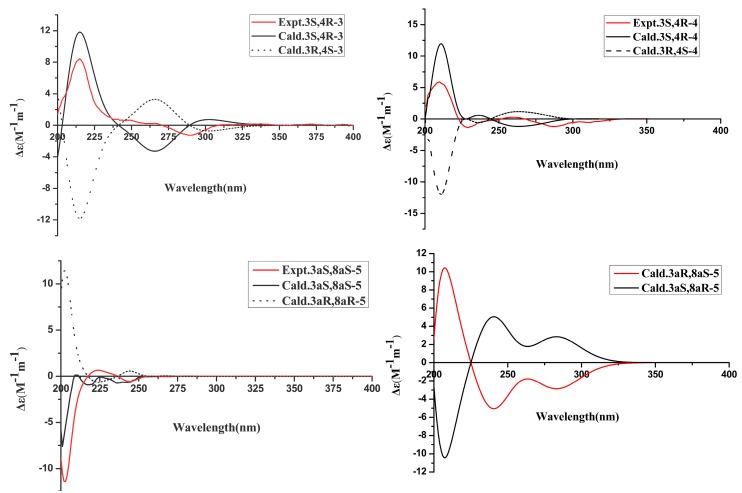
Calculated and experimental ECD spectra for compounds **3**, **4** and **5**.

The HRESIMS data of compound **3** at m/z 225.0757 ([M + H]^+^, calcd. C_11_H_13_O_5_ 225.0757) established the molecular formula C_11_H_12_O_5_. Its ^1^H NMR spectrum displayed a singlet for an aromatic methyl group (*δ*_H_ 2.09, s), a doublet for another methyl group (*δ*_H_ 1.18 s) attached to a methine, two phenolic hydroxyl groups (*δ*_H_ 11.27, s and 9.42, s), and a singlet for an aromatic proton (*δ*_H_ 6.68, s). The ^13^C NMR spectrum (Table 2) exhibited the existences of a benzene ring moiety (*δ*_c_ 162.7, 155.0, 146.5, 110.7, 101.3, 104.1), an ester carbonyl group (*δ*_c_ 171.5), two methyl (*δ*_c_ 17.8, 6.9) groups, and two oxygenated methine (*δ*c 84.2, 67.2) groups. These data indicated compound **3** was a dihydroisocoumarin derivative with great similarity to 4, 6-dihydroxymellein [12]. In view of the downfield chemical shift and the peak shape, the hydroxyl group at *δ*_H_ 11.27 must be attached to C-8 to form an intramolecular hydrogen bond with the carbonyl (C-1). Hence, the only difference between **3** and **4** is that 6-dihydroxymellein might be an additional methyl at C-7 in **3**. This conclusion was confirmed by the HMBC correlations from CH_3_-10 to C-6, C-7, and C-8, from 8-OH to C-7, C-8 and C-8a, and from H-5 to C-6, C-7, and C-8a. The deduction that CH_3_-9 and 4-OH facing *trans* direction was revealed by the strong nuclear Overhauser effect (NOE) correlation between H-4 and CH_3_-9. The absolute configuration of **3** was concluded to be 3*S*, 4*R,* in that the calculated ECD curve of (3*S*, 4*R*)-**3** had a good agreement with the experimental data (Figure 3).

The NMR features (Table 2) suggested that compound **4** was quite similar to **3**, except for two aromatic hydroxyl groups in **3** being replaced by two methoxy groups (*δ*_H_ 3.96, *δ*_c_ 55.8, 6-OCH_3_; *δ*_H_ 3.99, *δ*_c_ 61.2, 8-OCH_3_) in **4**, supported by the HMBC correlations from 6-OCH_3_ to C-6 and from 8-OCH_3_ to C-8, and by the molecular formula C_13_H_16_O_5_ according to HRESIMS at m/z 253.1078 ([M + H]^+^, calcd. C_13_H_17_O_5_ 253.1076). The positions of 6-OCH_3_ and 8-OCH_3_ were further confirmed by HMBC correlations from CH_3_-10 to C-6, C-7, and C-8, and from H-5 to C-6, C-7, and C-8a. The NOE correlation between H-4 and CH_3_-9, together with the similar experimental and calculated ECD curves shown in Figure 3, suggested that compound **4** had the same absolute configuration as **3**. Hence, compound **4** was elucidated as (3*S*, 4*R*)-4-hydroxy-6, 8-dimethoxy-3, 7-dimethyldihydroisocoumarin.

Compound **5** was obtained as a yellow solid. The molecular formula C_12_H_14_N_2_O_2_ (seven degrees of unsaturation) was established from HRESIMS at m/z 219.1127 ([M + H]^+^, calcd. C_12_H_15_N_2_O_2_ 219.1128). The IR spectrum showed an absorption band at 3366 cm^−1^, corresponding to an amino-group. In addition, the bands at 1615, 1457, and 1413 cm^−1^ were indicative of the amido carbonyl group and aromatic ring system. The ^13^C NMR (Table 3) and HSQC spectra of compound **5** revealed the presence of an acetyl methyl carbon (*δ*_C_ 22.0), one carbonyl carbon (*δ*_C_ 170.5), two sp^3^ hybridized methene carbons (*δ*_C_ 36.4, 47.0), four aromatic methine carbons (*δ*_C_ 130.7, 123.4, 119.5, 110.2), one heteroatom substituted methine carbon (*δ*_C_ 81.6), one heteroatom attached quaternary carbon (*δ*_C_ 86.6), and two phenolic quaternary carbons (*δ*_C_ 149.6, 129.2). These data were almost identical to those of the known compound (3a*R*, 8a*S*)-1-acetyl-1, 2, 3, 3a, 8, 8a-hexahydropyrrolo [2,3b] indol-3a-ol isolated from *Streptomyces staurosporeus* fermentation with tryptamine [13], which suggested that compound **5** had the same planar structure as the known compound. 

The deduction was further confirmed by detailed analysis of the HMBC correlations of **5** (Figure 2). Most chemical shifts of the ^1^H NMR data of **5** were also consistent with those of the known compound with differences ≤ 0.03 ppm in deuterated chloroform. However, the chemical shift of one of the H-3 protons at *δ*_H_ 2.70 in the latter was shifted upfield by 0.16 ppm to 2.54 ppm in the former (compound **5**), suggesting that the absolute configuration at C-3a in compound **5** might be different from that of the known compound. Moreover, the specific rotations of the two compounds greatly differed in value (**5**, ${\left[\alpha \right]}_{D}^{25}$ 23.06; the known compound, ${\left[\alpha \right]}_{D}^{25}$ 72.00; in MeOH), suggesting that they were a pair of diastereoisomers. Furthermore, NOE correlations between one of the protons of H-3 at *δ*_H_ 2.54 and the proton at *δ*_H_ 5.28, together with no NOE correlations between H-8a (*δ*_H_ 5.32) and the proton at *δ*_H_ 5.28, suggested that the proton at *δ*_H_ 5.28 should be 3a-OH, which was opposite to H-8a. Thus, the relative configuration of **5** was different from that of the known compound. On the basis of this deduction, the theoretical ECDs were calculated. From Figure 3, the experimental curve of compound **5** matched well with that of the calculative (3a*S*, 8a*S*)-**5** and greatly diverged from those of (3a*R*, 8a*R*)-**5,** (3a*R*, 8a*S*)-**5**, and (3a*S*, 8a*R*)-**5**, suggesting that the absolute configuration of **5** was 3a*S*, 8a*S*. Since 1-acetyl-1, 2, 3, 3a, 8, 8a-hexahydropyrrolo [2,3b] indol-3a-ol had been synthesized as a racemic mixture without specifying the relative configuration [14], it is impossible to judge whether compound **5** was a new compound or not. However, it can be concluded that this is the first report of **5** as a new natural product. Notably, as shown in the HPLC profiles (Figure S34), this new metabolite was produced after adding the ingredient (2*R*, 3*R*)-3, 5, 7-trihydroxyflavanone 3-acetate of the host [10], which exhibits weak antifungal activity against the research fungus L29, suggesting that the pressure to inhibit growth from the antifungal agent of the host changed the metabolic pathway of the fungus. To the best of our knowledge, this is the first example of a new natural product from plant endophytic fungus generated according to such a strategy.

Compounds **1**–**3** and **5** were evaluated in vitro for antifungal activity towards three phytopathogenic fungi: *Fusarium oxysporum* (*F. oxysporum*), *Penicillium italicum* (*P. italicum*), and *Fusarium graminearum* (*F. graminearum*) (Table 4). Compared to the positive control triadimefon, all of the compounds exhibited higher inhibitory activities against *F. oxysporum* and *F. graminearum* except for compound **1**. This compound displayed good activity against *F. oxysporum* (minimum inhibitory concentration (MIC) value = 112.6 *μ*M) and very weak activity towards *F. graminearum* (MIC value = 900.0 µM). The activities towards *P. italicum* for all compounds except compound **3** (inactive, MIC value > 900.0 µM), appeared to range between modest and high values (MIC values from 450.4 µM to 57.3 µM). It should be noted that compound **5** exhibited approximately twelve-, three-, and eighteen-fold stronger activities against *F. oxysporum*, *P. italicum,* and *F. graminearum* than triadimefon, respectively. Moreover, the activities on *F. oxysporum* for compounds **1** and **2**, and on *F. graminearum* for compounds **2** and **3**, were approximately three and two point five times higher than those of triadimefon, respectively. The activities of compound **4** were not assessed because the amount produced was too small.

## 3. Materials and Methods

### 3.1. General Experimental Procedures

Optical rotations were recorded using a P-1020 digital polarimeter (Jasco International Co., Ltd., Tokyo, Japan). Ultraviolet (UV) and IR spectra were obtained using a UV-2550 spectrophotometer (Shimadzu Corporation, Tokyo, Japan) and Nicolet iS10 Fourier transform infrared spectrophotometer (Thermo Electron Corporation, Madison, WI, USA), respectively. CD spectra were collected on Chirascan CD spectrometer (Applied Photophysics Ltd., London, UK). ^1^H and ^13^C NMR data were collected on an AVIII 600 MHz NMR spectrometer (Bruker BioSpin GmbH Company, Rheinstetten, Germany). The positive ion mode on a quadrupole-time of flight (Q-TOF) mass spectrometer (Thermo Fisher Scientific Inc., Frankfurt, Germany) was used for the measurement of HRESIMS. Semi-preparative HPLC was performed on a 1260 Infinity Series high performance liquid chromatography system (Agilent Corporation, Santa Clara, CA, USA). Common column and thin layer chromatography (TLC) was performed using a 200−300 mesh and G60, F-254 silica gel (Qingdao Haiyang Chemicals Co., Ltd., Qingdao, China), respectively. For gel filtration chromatography, a Sephadex LH-20 (GE Healthcare, Chicago, IL, USA) was used.

### 3.2. Fungal and Plant Material

*M. bontioides* was collected from a mangrove in Leizhou Peninsula, China, in May 2014. The strain L29 was obtained from its leaf and was identified as *Botryosphaeria ramosa* on the basis of molecular analysis methods [15]. These cultures are deposited in the College of Materials and Energy, South China Agricultural University. A BLAST research by NCBI showed that the internal transcribed spacer (ITS) sequence (No. MK370738 in GenBank) of the fungal strain was identical to that of *Botryosphaeria ramosa* CMW26167 (No. NR151841.1).

### 3.3. Cultivation, Extraction, and Isolation

The strain maintained on potato dextrose agar (PDA) medium at 28 °C for 3 days was put into the liquid medium (2% glucose, 2% peptone) and incubated at 28 °C for about 4 days. After that, the culture (6 mL) was transferred to an Erlenmeyer flask with the rice medium (70 mL H_2_O, 50 g rice, 0.25 g crude sea salt) treated either with (2*R*, 3*R*)-3, 5, 7-trihydroxyflavanone 3-acetate (0.25 mM) or without this compound (control), and was incubated for 28 days at room temperature. The flavanone was isolated from *M. bontioides*, the host of the fungus *B. ramosa* L29. It was shown to cause an 18.56% growth inhibition rate at the concentration of 0.25 mM to the mycelium of *B. ramosa* L29 by a petri plate mycelia growth rate method [16]. The media (20 bottles) were continuously extracted three times using 95% ethanol. The solvent was evaporated in vacuo and was extracted three times with ethyl acetate to obtain a dark brown crude extract (25.0 g). The EtOAc extracts of different conditions were analyzed by reversed-phase HPLC (Hypersil BDS C18 column, 150 × 4.6 mm, 5 µm) using a gradient of MeOH/H_2_O (20:80–80:20, 0–30 min; 80:20–100: 0, 30–45 min; 100: 0, 45–60 min) at a flow rate of 1.0 mL/min, and recorded at 254 nm. Then, the EtOAc extract was subjected to silica gel column chromatography eluted with a gradient of petroleum ether/EtOAc (90:10, 85:15, 75:25, 50:50, 25: 75, 15:80, *v*/*v*) to afford six fractions (Fraction 1–6). Fraction 1 was chromatographed on a silica gel column, eluted with a gradient system of petroleum ether/EtOAc (95:5, 90:10, 85:15, *v/v*) to provide Fraction 1.1–1.5; Fraction 1.2 was separated through a Sephadex LH-20 chromatograph (MeOH as eluent) to give Fraction 1.1.1–1.1.2, and Fraction 1.1.1 was purified through semi-preparative HPLC (MeOH/H_2_O,90:10, *v*/*v*; 2.0 mL/min) to yield compound **2** (3 mg, t_R_ = 33.5 min). Fraction 2 was eluted with a gradient system of petroleum ether/EtOAc (90:10, 85:15, 80:20, 75:25, *v*/*v*) on a silica gel column to give Fraction 2.1–2.4; Fraction 2.2 was applied on semi-preparative HPLC (MeOH/H_2_O, 79:21, *v*/*v*; 2.0 mL/min) to provide compound **4** (1.5 mg, t_R_ = 29.3 min). Then, Fraction 3 was fractioned by silica gel column chromatography using petroleum ether/ EtOAc (80:20, 75:25, 70:30, 60:40, 50:50, 40:60, 30:70,20:80, *v*/*v*) as eluents to afford Fraction 3.1–3.8; Fraction 3.4 was subjected to semi-preparative HPLC (MeOH/H_2_O, 68:32, *v*/*v*; 2.0 mL/min) to yield compounds **3** (2.8 mg, t_R_ = 22.1 min) and **1** (2.5 mg, t_R_ = 25.2 min). Further separation of Fraction 3.7 by semi-preparative HPLC (MeOH/H_2_O, 65:35, *v*/*v*; 2.0 mL/min) led to the isolation of compound **5** (2 mg, t_R_ = 20.4 min).

Compound **1**. Yellow powder; IR (KBr) *ν*_max_ 3244, 2940, 1682, 1622, 1153, 1164, 1615, 834 cm^−1^; HRESIMS m/z 223.0608 ([M + H]^+^, calcd. for C_11_H_11_O_5_ 223.0606); ^13^C NMR and ^1^H NMR (Table 1).

Compound **2**. Colorless powder; HRESIMS m/z 237.0765 ([M + H]^+^, calcd. for C_12_H_13_O_5_ 237.0763); ^13^C NMR and ^1^H NMR (Table 1).

Compound **3**. Colorless powder; UV (CH_3_CN) *λ*_max_ (log *ε*) 217 (1.97), 261 (0.68), 298 (0.22) nm; ${\left[\alpha \right]}_{D}^{25}$ 16.80 (c 0.30, MeOH); HRESIMS m/z 225.0757 ([M + H]^+^, calcd. for C_11_H_13_O_5_ 225.0757); ^13^C NMR and ^1^H NMR (Table 2).

Compound **4**. Colorless powder; UV (CH_3_CN) *λ*_max_ (log *ε*) 217 (0.90), 260 (0.31) nm; ${\left[\alpha \right]}_{D}^{25}$ 16.00 (c 0.25, MeOH); HRESIMS m/z 253.1078 ([M + H]^+^, calcd. for C_13_H_17_O_5_ 253.1076); ^13^C NMR and ^1^H NMR (Table 2).

Compound **5**. Yellow solid; UV (CH_3_CN) *λ*_max_ (log *ε*) 204 (2.26), 234 (0.97), 297 (0.17) nm; IR (KBr) *ν*_max_ 3366, 2922, 1615, 1457, 1413, 1313, 1188, 1061, 752 cm^−1^; ${\left[\alpha \right]}_{D}^{25}$ 23.06 (c 0.19, MeOH); HRESIMS m/z 219.1127 ([M + H]^+^, calcd. for C_12_H_15_O_2_N_2_ 219.1128); ^13^C NMR and ^1^H NMR (Table 3).

### 3.4. ECD Calculations

Conformational analysis of the enantiomers of compounds **3**–**5** established by NOESY analyses were carried out via searching with the MMFF94s molecular mechanics force field using Spartan’10 software (Wavefunction Inc., Irvine, CA, USA) within 10 kcal/mol. The Gaussian 09 program (Gaussian Inc., Wallingford, CT, USA) was used to optimize by density functional theory (DFT) at the 6-31G (d, p) level (methanol as the solvent), and to calculate ECD on 6-311 + G(d, p) level by time-dependent density functional theory (TDDFT). Boltzmann statistics were performed for ECD simulations with a standard deviation of 0.3 eV. The softwares SpecDis 1.64 (University of Wurzburg, Wurzburg, Germany) and OriginPro 8.5 (OriginLab, Ltd., Northampton, MA, USA) were used to generate the ECD curves.

### 3.5. Antifungal Activity Assay

Three fungi including *F. oxysporum*, *P. italicum*, and *F. graminearum* used for bioassay were acquired from the College of Agriculture, South China Agricultural University. The antifungal effects were examined by the two-fold broth dilution method, as previously reported [17].

## 4. Conclusions

In this study, four new isocoumarin derivatives, which we named botryospyrones A, B, C, and D, along with a new natural tryptamine derivative, (3a*S*, 8a*S*)-1-acetyl-1, 2, 3, 3a, 8, 8a-hexahydropyrrolo [2,3b] indol-3a-ol, were isolated from an endophytic fungus *B. ramosa* L29, obtained from the leaf of *M. bontioides*. Compound **1** remarkably inhibited *F. oxysporum*, compounds **2** and **3** highly inhibited *F. oxysporum* and *F. graminearum*, and compound **5** significantly inhibited *F. oxysporum*, *P. italicum*, and *F. graminearum*. The compounds were more potent than triadimefon, revealing their potential to be used as new antifungal leads. Compound **5** was produced when using the “UISCH” strategy by adding (2*R*, 3*R*)-3, 5, 7-trihydroxyflavanone 3-acetate, a component of *M. bontioides* with weak growth inhibitory activity towards *B. ramosa* L29, into the medium, suggesting that this strategy could be applied to induce an endophyte to produce new bioactive molecules.

## Figures and Tables

**Table 1:** ^1^H (600MHz) and ^13^C NMR (150MHz) data for compounds **1** and **2**.

Position	1 ^a^		2 ^a^	
	*δ*_H_, multi (*J*/Hz)	*δ*c (ppm)	*δ*_H_, multi (*J*/Hz)	*δ*c (ppm)
1		166.7		166.4
3		153.7		153.9
4	6.38,s	101.6	6.51,d(1.0)	99.0
4a		137.6		130.7
5		108.7		134.7
6		161.6		159.8
7		128.6	6.50,s	98.6
8		161.4		160.0
8a		99.8		98.3
5-OCH_3_			3.93,s	61.4
6-OCH_3_			3.78,s	56.1
6-OH	5.38,s			
7-OH	6.42,s			
8-OH	11.18,s		10.99,s	
9	2.29,s	19.6	2.27,d(1.0)	19.6
10	2.18,s	9.8		

^a^ Measured in CDCl_3_.

**Table 2:** ^1^H (600MHz) and ^13^C NMR (150MHz) data for compounds **3** and **4**.

Position	3 ^a^		4 ^a^	
	*δ*_H_, multi (*J*/Hz)	*δ*c	*δ*_H_, multi (*J*/Hz)	*δ*c
1		171.5		166.5
3	5.33,d(4.8)	84.2	5.30,d(3.0)	82.5
4	4.17,m	67.2	4.29,m	67.2
4a		146.5		149.8
5	6.68,s	101.3	7.00,s	99.8
6		155.0		163.8
7		110.7		119.5
8		162.7		157.1
8a		104.1		110.8
6-OH	9.42,s			
8-OH	11.27,s			
6-OCH_3_			3.96,s	55.8
8-OCH_3_			3.99,s	61.2
9	1.18,d(4.8)	17.8	1.22,d(6.6)	18.1
10	2.09,s	6.9	2.11,s	7.8

^a^ Measured in (CD_3_)_2_CO.

**Table 3:** ^1^H (600MHz) and ^13^C NMR (150MHz) data for compound **5**.

Position	5 ^a^	
	*δ*_H_, multi (*J*/Hz)	*δ*_C_
2	3.30,m(10.2,6.6)	47.0
	3.71,m(10.2,3.0)	
3	2.47,m	36.4
	2.54,m	
3a		86.6
3b		129.2
4	7.30,d(7.2)	123.4
5	6.81,dd (7.8,7.2)	119.5
6	7.18,dd(7.8,7.2)	130.7
7	6.64,d(7.8)	110.2
7a		149.6
8	-NH unobserved	
8a	5.32,s	81.6
9		170.5
10	2.03,s	22.0
3a-OH	5.28,s	

^a^ Measured in CDCl_3_.

**Table 4:** Antifungal activity of compounds **1**, **2**, **3**, and **5** measured as minimum inhibitory concentration (MIC) values.

Compounds	*F. oxysporum*	*P. italicm*	*F. graminearum*
	MIC, µM		
**1**	112.6	450.4	900.0
**2**	105.8	211.7	211.7
**3**	223.0	>900.0	223.0
**5**	28.6	57.3	28.6
Triadimefon ^a^	340.4	170.2	510.7

^a^ Positive control towards the test fungi.

## Supplementary Materials

The following are available online at http://www.mdpi.com/1660-3397/17/2/88/s1, Figures S1–S34: the NMR and HRESIMS spectra for all the isolated compounds, UV spectra for compounds **3**–**5**, IR spectra for compounds **1** and **5**, HPLC profiles for *B. ramosa* L29 EtOAc extract cultured in autoclaved rice medium with/without 0.25 mM (2*R*, 3*R*)-3, 5, 7-trihydroxyflavanone 3-acetate.
