# Supplementary material for: Four New Isocoumarins and a New Natural Tryptamine with Antifungal Activities from a Mangrove Endophytic Fungus Botryosphaeria ramosa L29
Source: Mar Drugs. 2019 Feb 1;17(2):88. doi: 10.3390/md17020088 (PMC6410081; doi:10.3390/md17020088)
Supplement: Supplementary file 1 [file marinedrugs-17-00088-s001.pdf]

Supplementary Materials

Four New Isocoumarins and a New Natural Tryptamine with Antifungal Activities from a Mangrove Endophytic Fungus *Botryosphaeria ramosa* L29

Zhihui Wu [1], Jiaqing Chen [1], Xiaolin Zhang [1], Zelin Chen [1], Tong Li [1], Zhigang She [2], Weijia Ding [1,*] and Chunyuan Li [1,*]

[1] College of Materials and Energy, South China Agricultural University, Guangzhou 510642, China; w_zhi_hui@sina.com (Z.W.); ch_jiaqing@sina.com (J.C.); catherinezxll@sina.com (X.Z.); czrin@sina.cn (Z.C.); zjjlitong@sina.com (T.L.); dwjzsu@163.com (W.D.); chunyuanli@scau.edu.cn (C.L.)

[2] School of Chemistry, Sun Yat-Sen University, Guangzhou 510275, China; cesshzhg@mail.sysu.edu.cn (Z.S.)

* Correspondence: dwjzsu@163.com (W.D.); chunyuanli@scau.edu.cn (C.L.); Tel.: +86-020-8528-0319 (W.D. and C.L.)

Contents

Figure S1 ^{1}H NMR spectrum (600 MHz) of compound **1** in $CDCl_3$ 3
Figure S2 ^{13}C NMR spectrum (150 MHz) of compound **1** in $CDCl_3$ 3
Figure S3 HSQC spectrum (600/150 MHz) of compound **1** in $CDCl_3$ 4
Figure S4 HMBC spectrum (600/150 MHz) of compound **1** in $CDCl_3$ 4
Figure S5 HRESIMS spectrum of compound **1** 5
Figure S6 IR spectrum of compound **1** 5
Figure S7 ^{1}H NMR spectrum (600 MHz) of compound **2** in $CDCl_3$ 6
Figure S8 ^{13}C NMR spectrum (150 MHz) of compound **2** in $CDCl_3$.......... 6
Figure S9 HSQC spectrum (600/150 MHz) of compound **2** in $CDCl_3$ 7
Figure S10 HMBC spectrum (600/150 MHz) of compound **2** in $CDCl_3$ 7
Figure S11 HRESIMS spectrum of compound **2** 8
Figure S12 ^{1}H NMR spectrum (600 MHz) of compound **3** in $(CD_3)_2CO$ 8
Figure S13 ^{13}C NMR spectrum (150 MHz) of compound **3** in $(CD_3)_2CO$ 9
Figure S14 HSQC spectrum (600/150 MHz) of compound **3** in $(CD_3)_2CO$ 9
Figure S15HMBC spectrum (600/150 MHz) of compound **3** in $(CD_3)_2CO$.......... 10
Figure S16 NOESY spectrum (600/150 MHz) of compound **3** in $(CD_3)_2CO$ 10
Figure S17 HRESIMS spectrum of compound **3** 11
Figure S18 UV spectrum of compound **3**.......... 11
Figure S19 ^{1}H NMR spectrum (600 MHz) of compound **4** in $(CD_3)_2CO$ 12
Figure S20 ^{13}C NMR spectrum (150 MHz) of compound **4** in $(CD_3)_2CO$ 12
Figure S21 HSQC spectrum (600/150 MHz) of compound **4** in $(CD_3)_2CO$ 13
Figure S22 HMBC spectrum (600/150 MHz) of compound **4** in $(CD_3)_2CO$.......... 13
Figure S23 NOESY spectrum (600/150 MHz) of compound **4** in $(CD_3)_2CO$ 14
Figure S24 HRESIMS spectrum of compound **4** 14
Figure S25 UV spectrum of compound **4**.......... 15
Figure S26 ^{1}H NMR spectrum (600 MHz) of compound **5** in $CDCl_3$ 15
Figure S27 ^{13}C NMR spectrum (150 MHz) of compound **5** in $CDCl_3$ 16
Figure S28 HMQC spectrum (600/150 MHz) of compound **5** in $CDCl_3$ 16
Figure S29 HMBC spectrum (600/150 MHz) of compound **5** in $CDCl_3$ 17
Figure S30 NOESY spectrum (600/150 MHz) of compound **5** in $CDCl_3$ 17
Figure S31 HRESIMS spectrum of compound **5** 18
Figure S32 UV spectrum of compound **5** 18
Figure S33 IR spectrum of compound **5** 19
Figure S34 (a) HPLC profile for *B. ramosa* L29 EtOAc extract cultured in autoclaved rice medium; (b) HPLC profile of *B. ramosa* L29 EtOAc extract cultured in autoclaved rice medium with 0.25 mM (2*R*, 3*R*)-3, 5, 7-trihydroxyflavanone 3-acetate from *M. bontioides*.......... 20

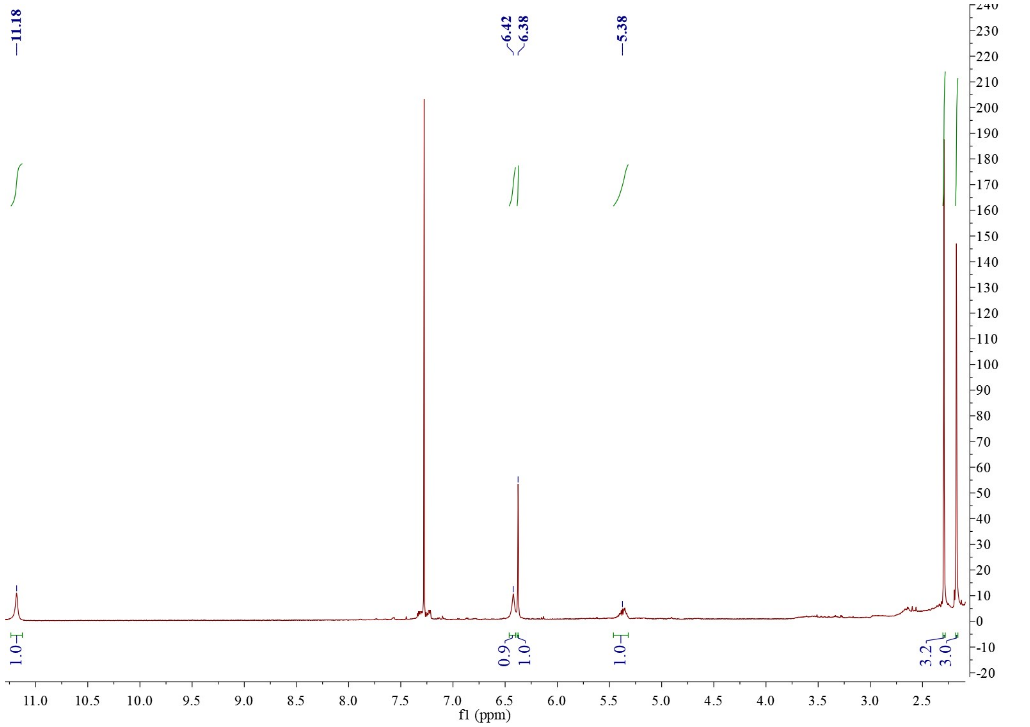

Figure S1 ^{1}H NMR spectrum (600 MHz) of compound **1** in $CDCl_3$

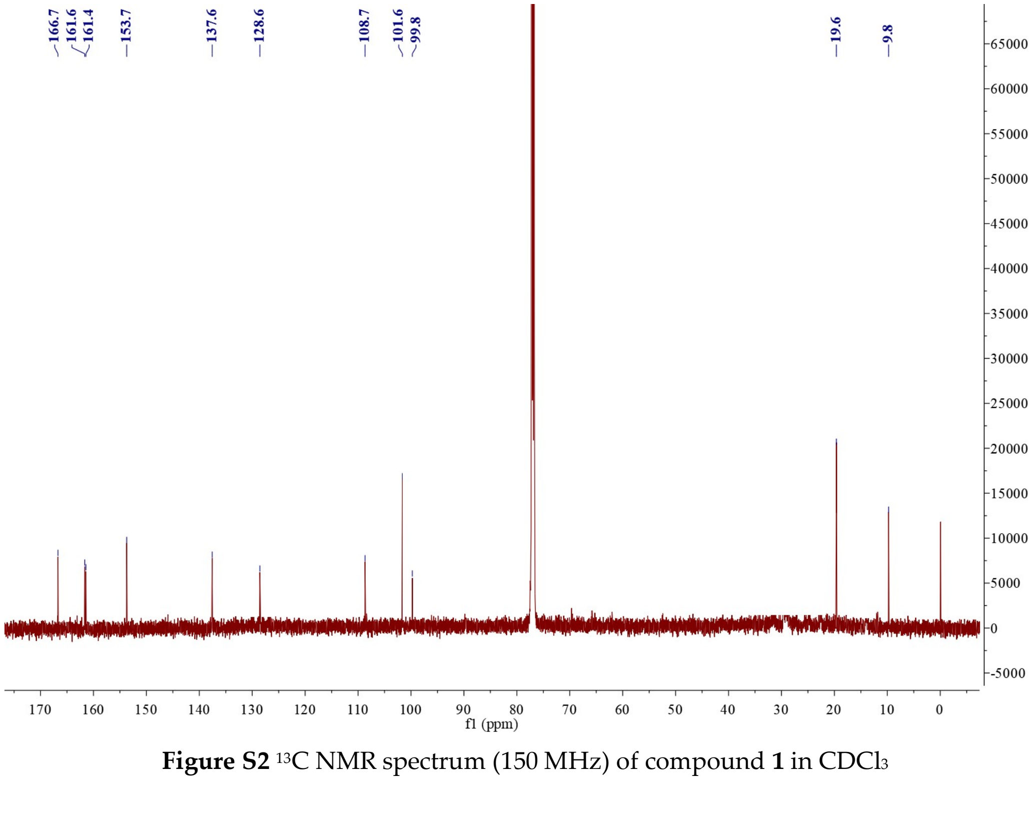

Figure S2 ^{13}C NMR spectrum (150 MHz) of compound **1** in $CDCl_3$

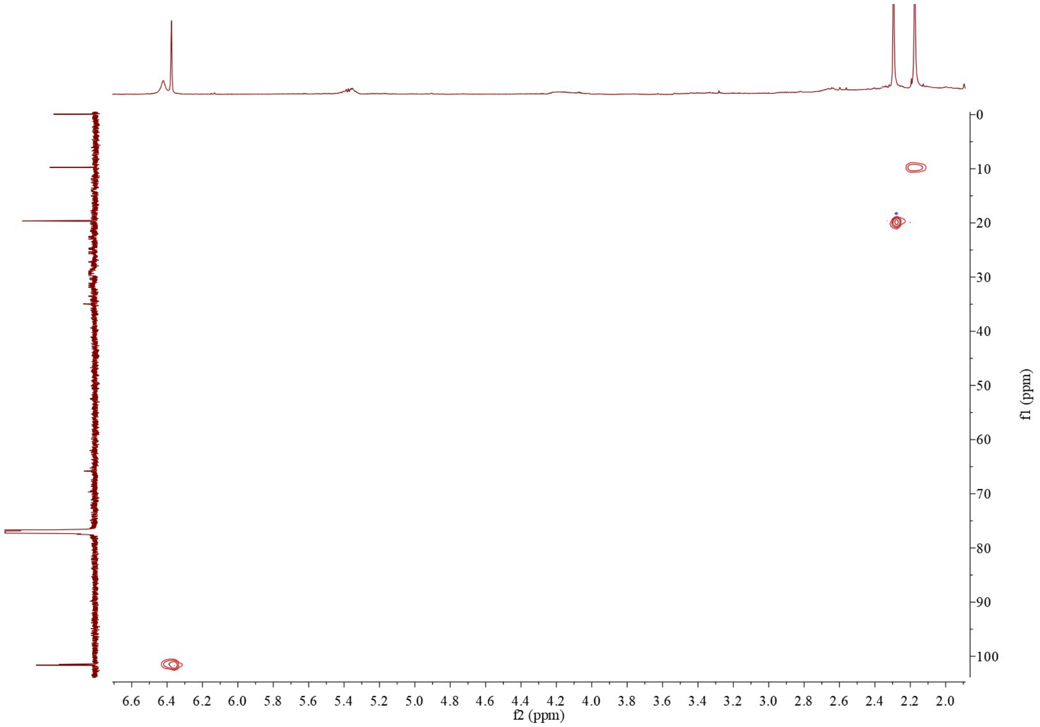

Figure S3 HSQC spectrum (600/150 MHz) of compound **1** in $CDCl_3$

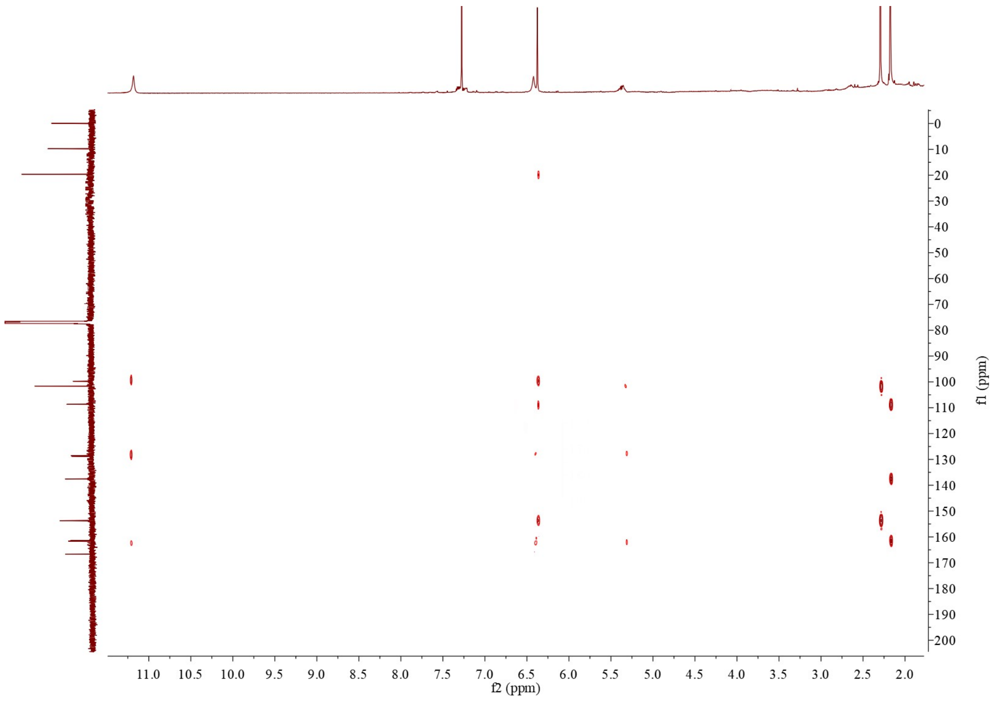

Figure S4 HMBC spectrum (600/150 MHz) of compound **1** in $CDCl_3$

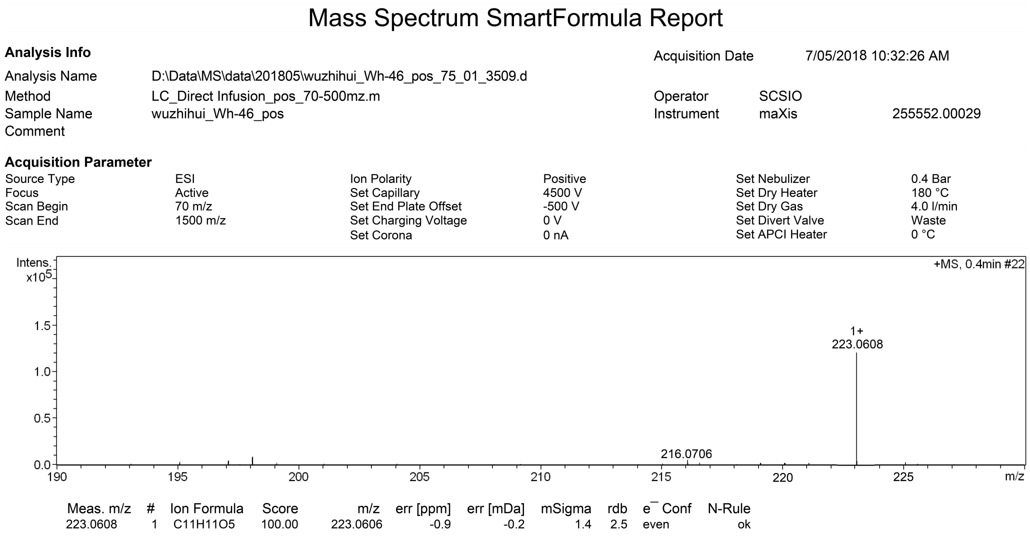

Figure S5 HRESIMS spectrum of compound **1**

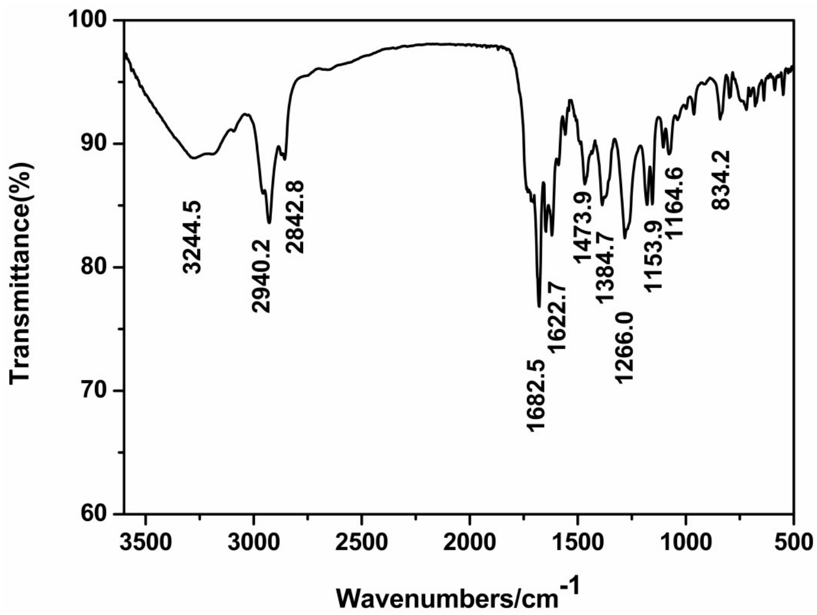

Figure S6 IR spectrum of compound **1**

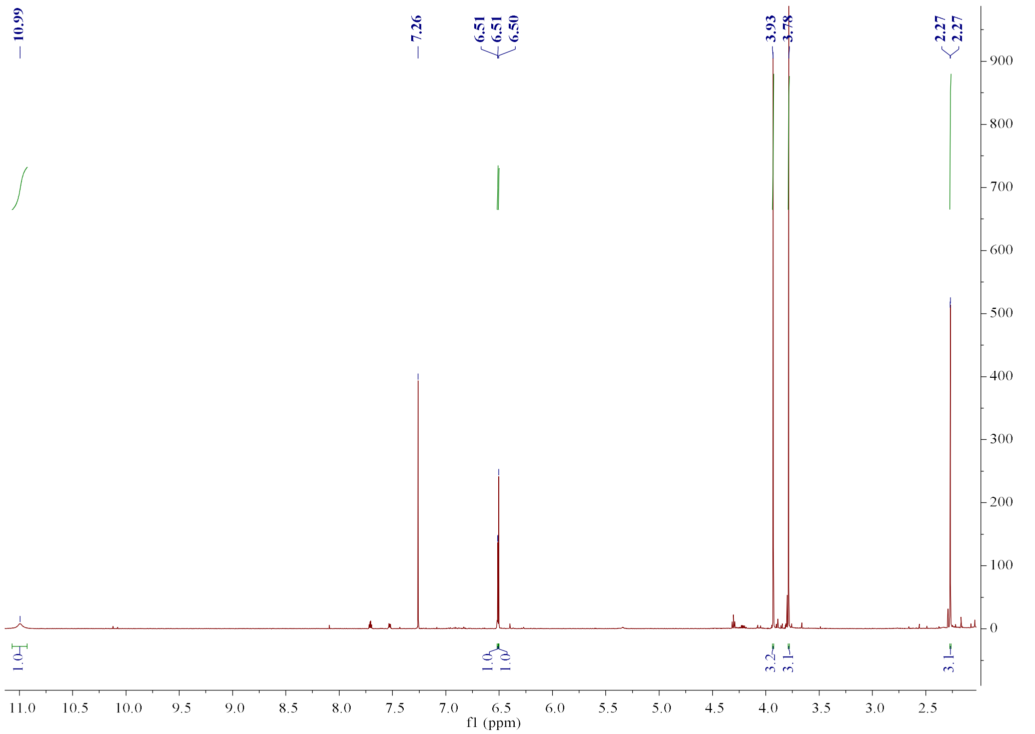

Figure S7 ^{1}H NMR spectrum (600 MHz) of compound **2** in $CDCl_3$

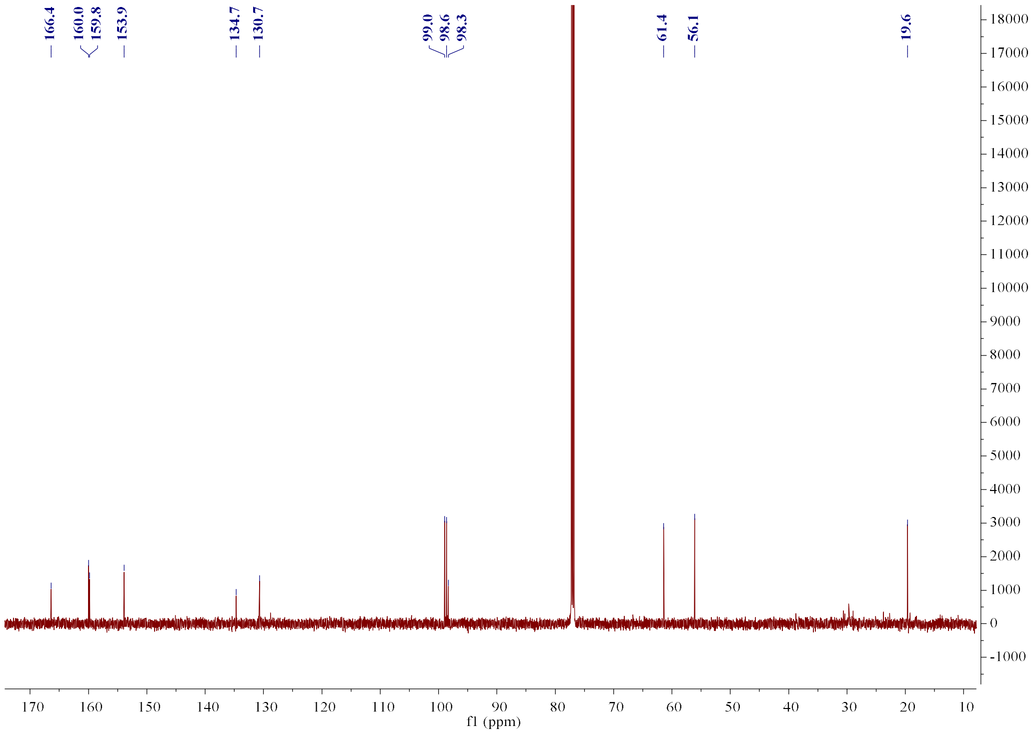

Figure S8 ^{13}C NMR spectrum (150 MHz) of compound **2** in $CDCl_3$

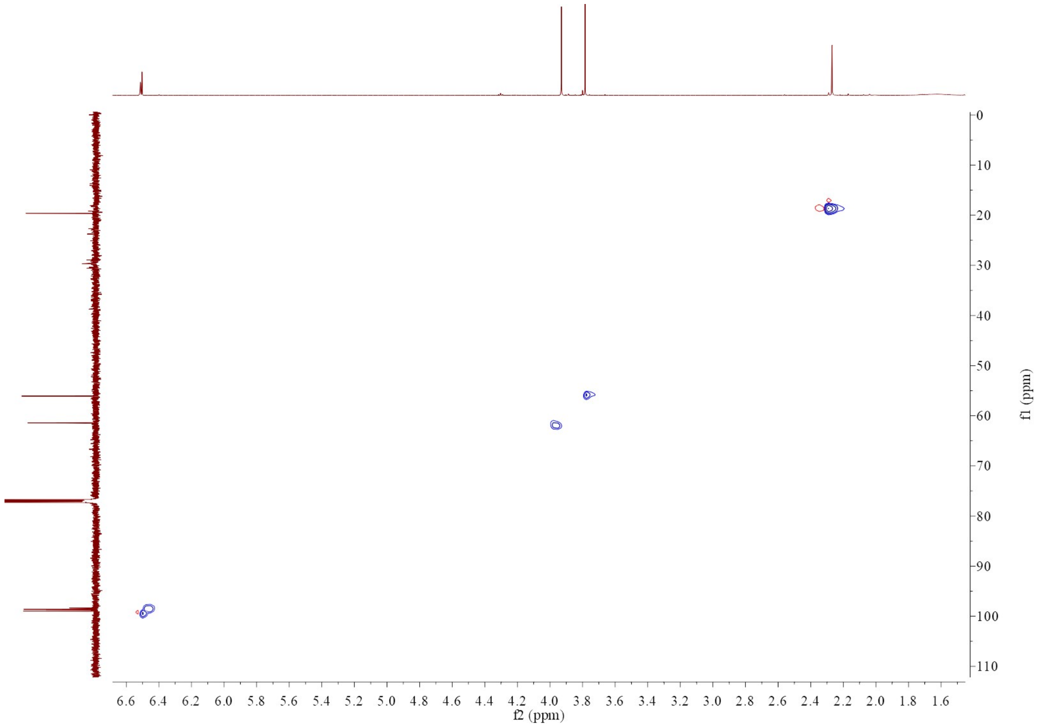

Figure S9 HSQC spectrum (600/150 MHz) of compound **2** in $CDCl_3$

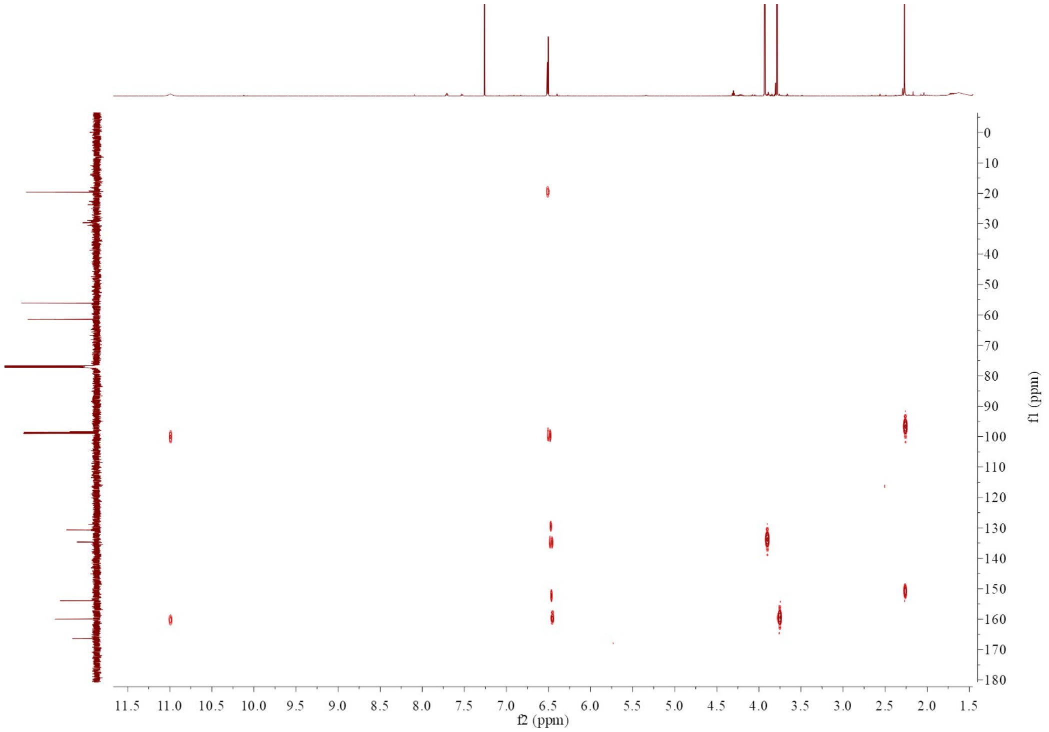

Figure S10 HMBC spectrum (600/150 MHz) of compound **2** in $CDCl_3$

Mass Spectrum SmartFormula Report

Analysis Info

Analysis Name	D:\Data\MS\data\201805\wuzh_wh39_pos_13_01_2966.d	Acquisition Date	5/4/2018 4:57:46 PM
Method	LC_Direct Infusion_pos_100-1000mz.m	Operator	SCSIO
Sample Name	Wh-39-1	Instrument	255552.00029
Comment			

Acquisition Parameter

Source Type	ESI	Ion Polarity	Positive	Set Nebulizer	0.4 Bar
Focus	Active	Set Capillary	4500 V	Set Dry Heater	180 °C
Scan Begin	100 m/z	Set End Plate Offset	-500 V	Set Dry Gas	4.0 l/min
Scan End	2000 m/z	Set Charging Voltage	0 V	Set Divert Valve	Waste
		Set Corona	0 nA	Set APCI Heater	0 °C

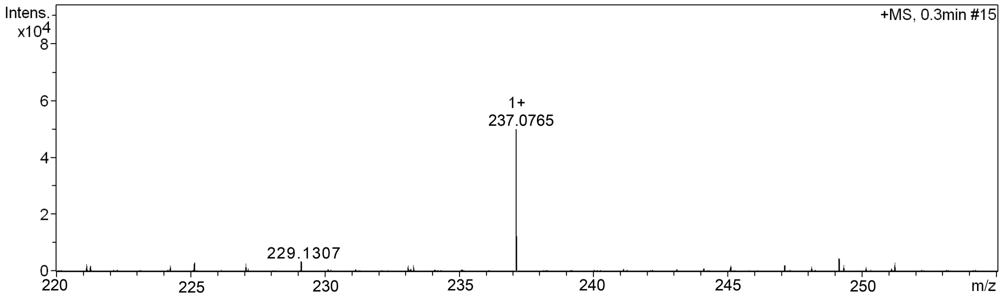

Meas. m/z	#	Ion Formula	Score	m/z	err [ppm]	err [mDa]	mSigma	rdb	e¯ Conf	N-Rule
237.0765	1	C12H13O5	100.00	237.0763	0.5	0.2	1.3	9.5	even	ok

Figure S11 HRESIMS spectrum of compound **2**

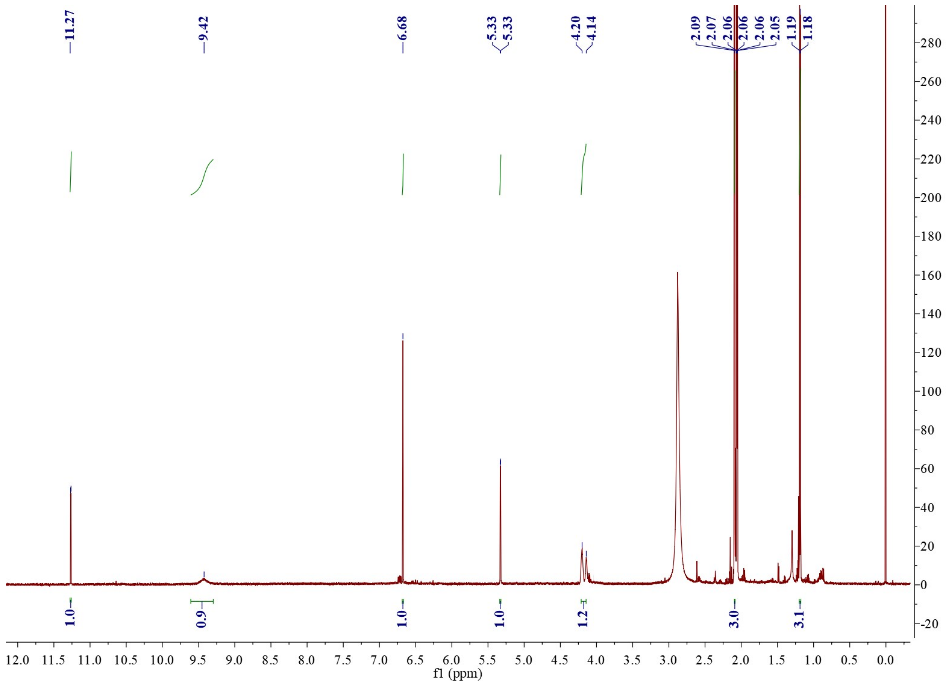

Figure S12 ^{1}H NMR spectrum (600 MHz) of compound **3** in $(CD_3)_2CO$

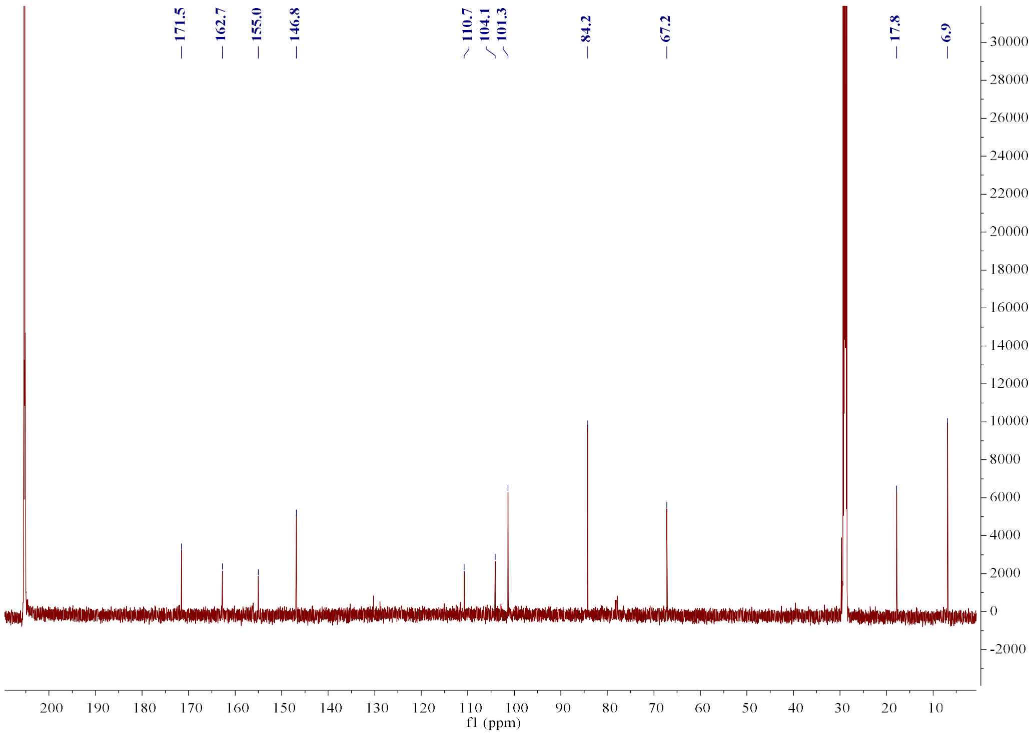

Figure S13 ^{13}C NMR spectrum (150 MHz) of compound **3** in $(CD_3)_2CO$

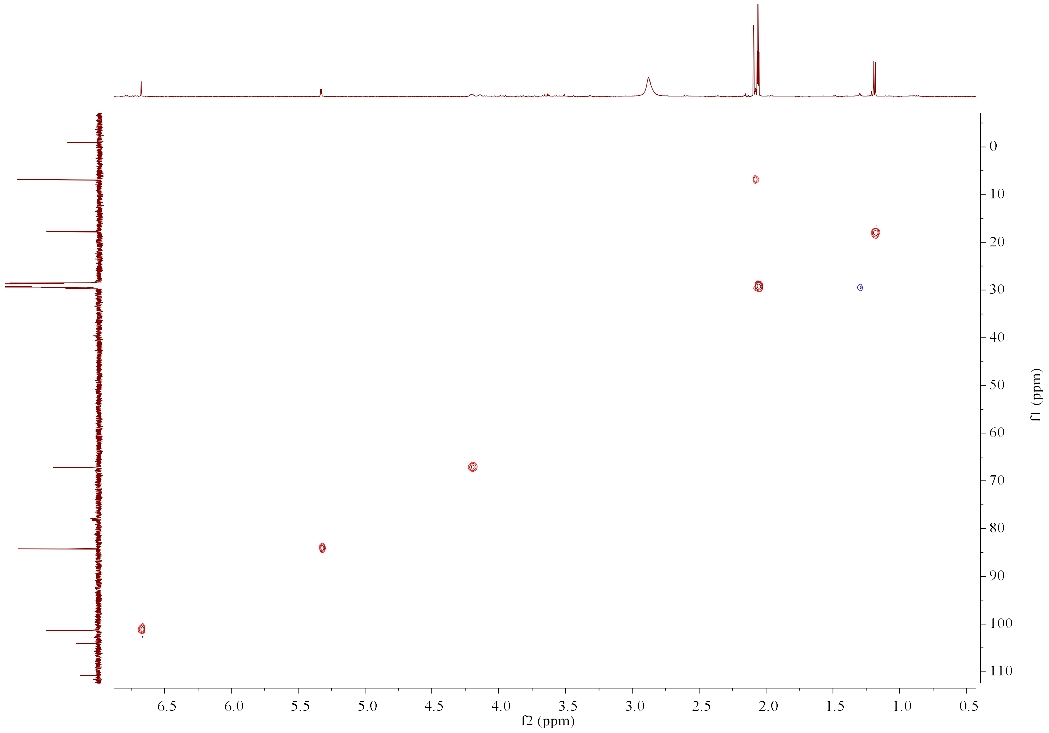

Figure S14 HSQC spectrum (600/150 MHz) of compound **3** in $(CD_3)_2CO$

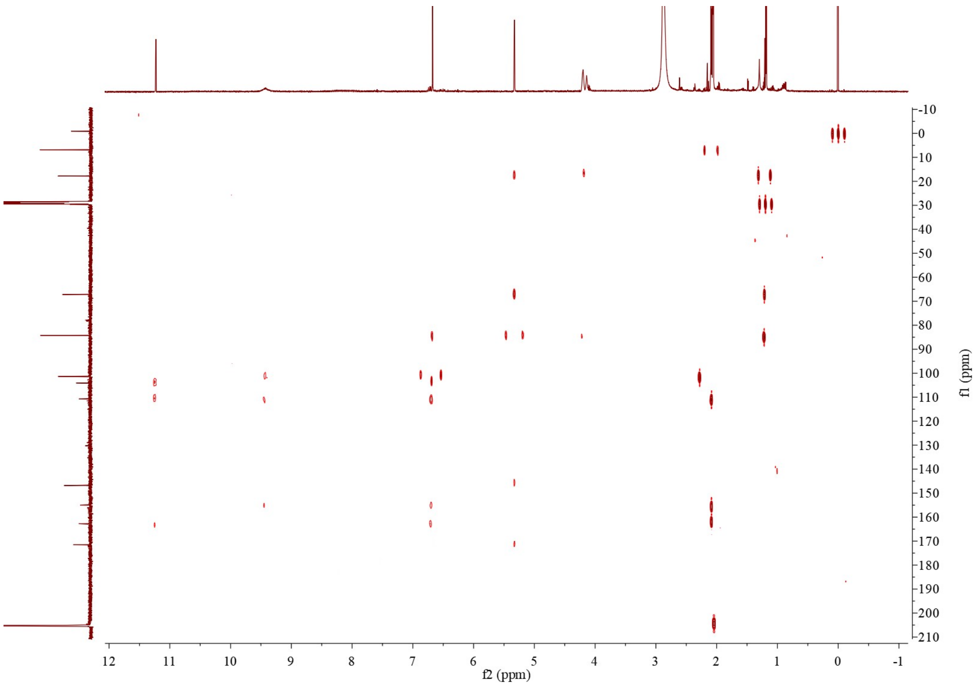

Figure S15 HMBC spectrum (600/150 MHz) of compound **3** in $(CD_3)_2CO$

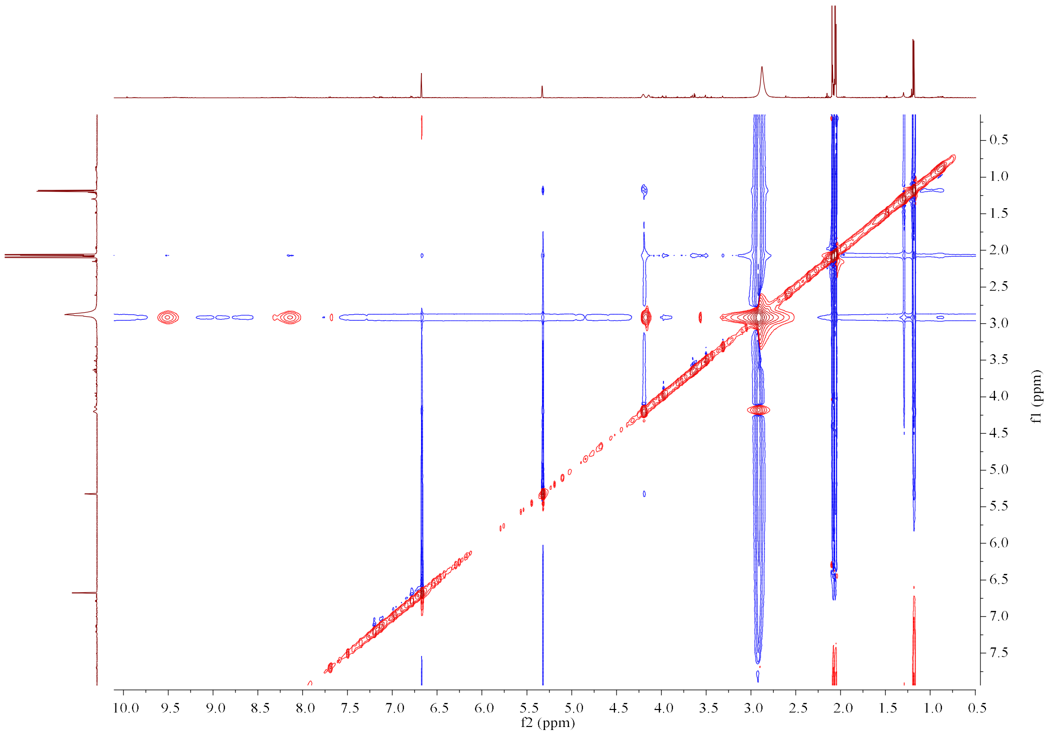

Figure S16 NOESY spectrum (600/150 MHz) of compound **3** in $(CD_3)_2CO$

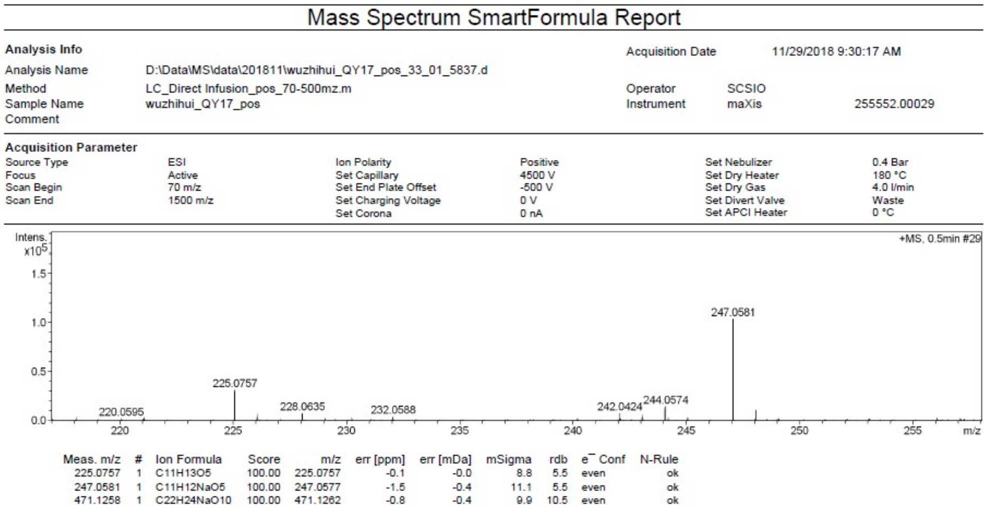

Figure S17 HRESIMS spectrum of compound **3**

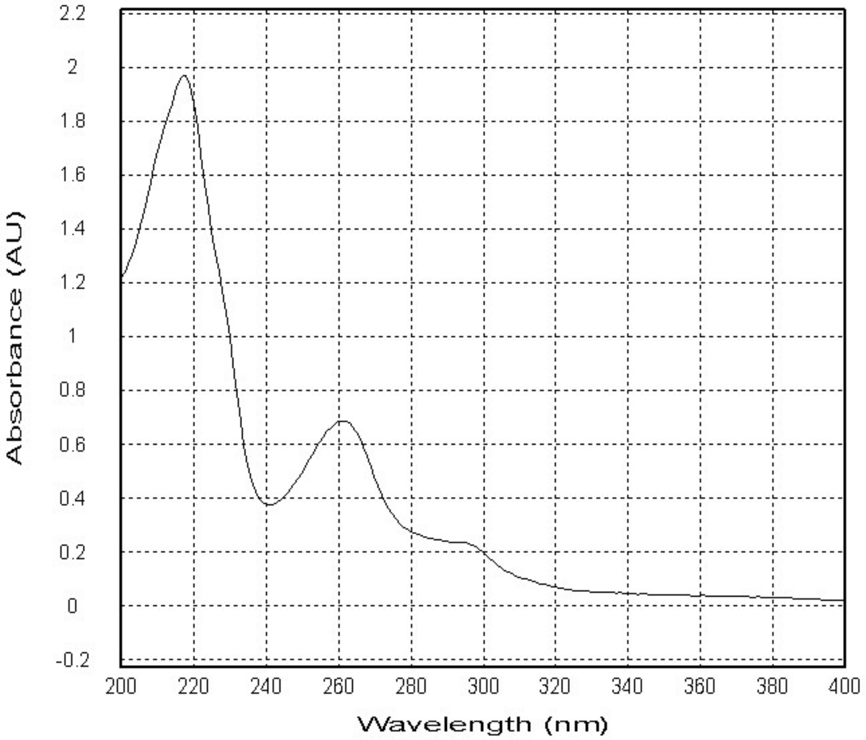

Figure S18 UV spectrum of compound **3**

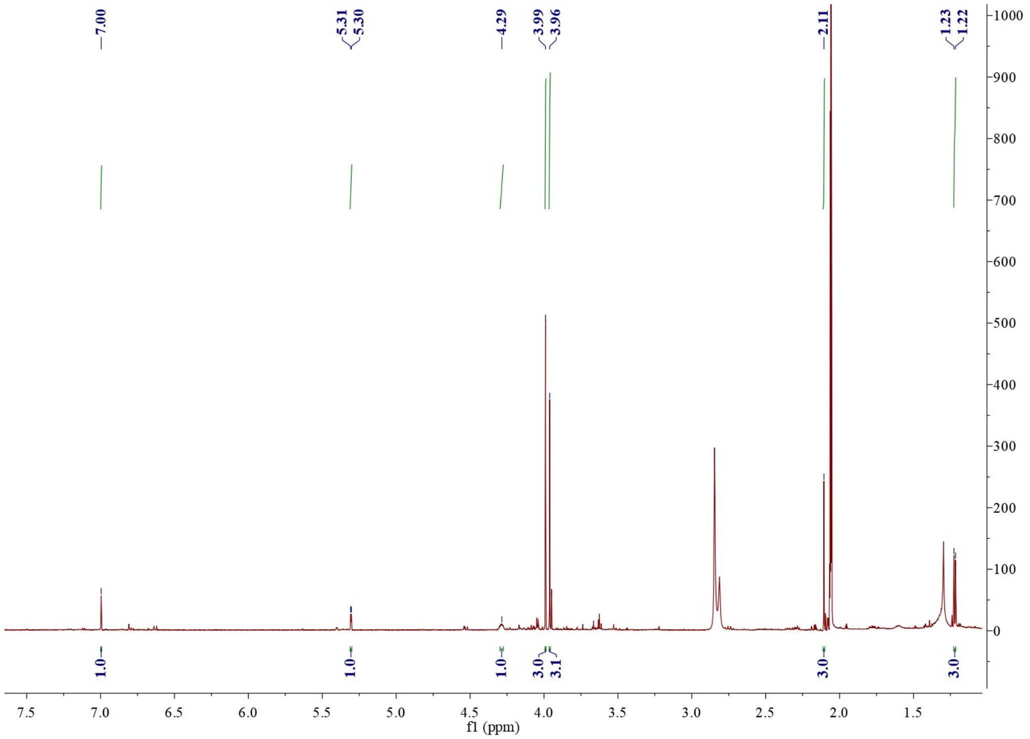

Figure S19 ^{1}H NMR spectrum (600 MHz) of compound **4** in $(CD_3)_2CO$

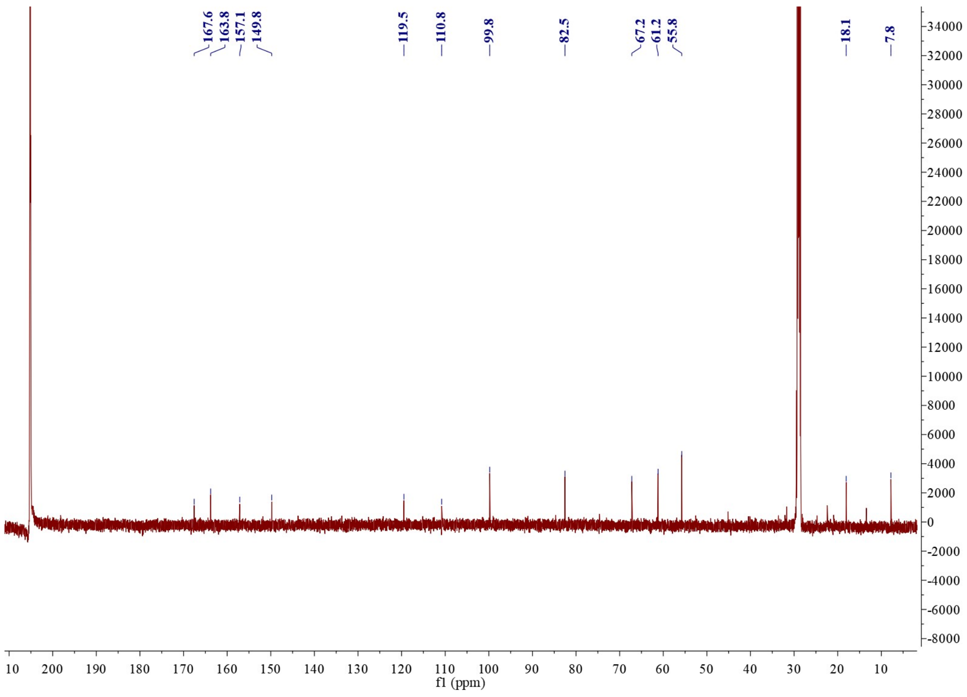

Figure S20 ^{13}C NMR spectrum (150 MHz) of compound **4** in $(CD_3)_2CO$

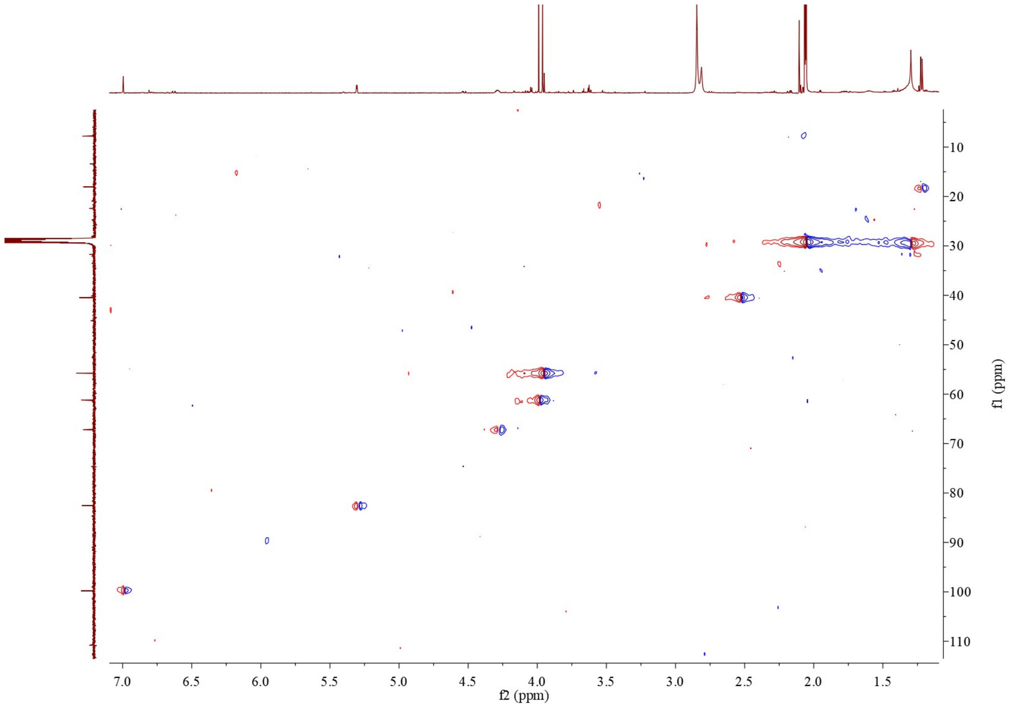

Figure S21 HSQC spectrum (600/150 MHz) of compound **4** in $(CD_3)_2CO$

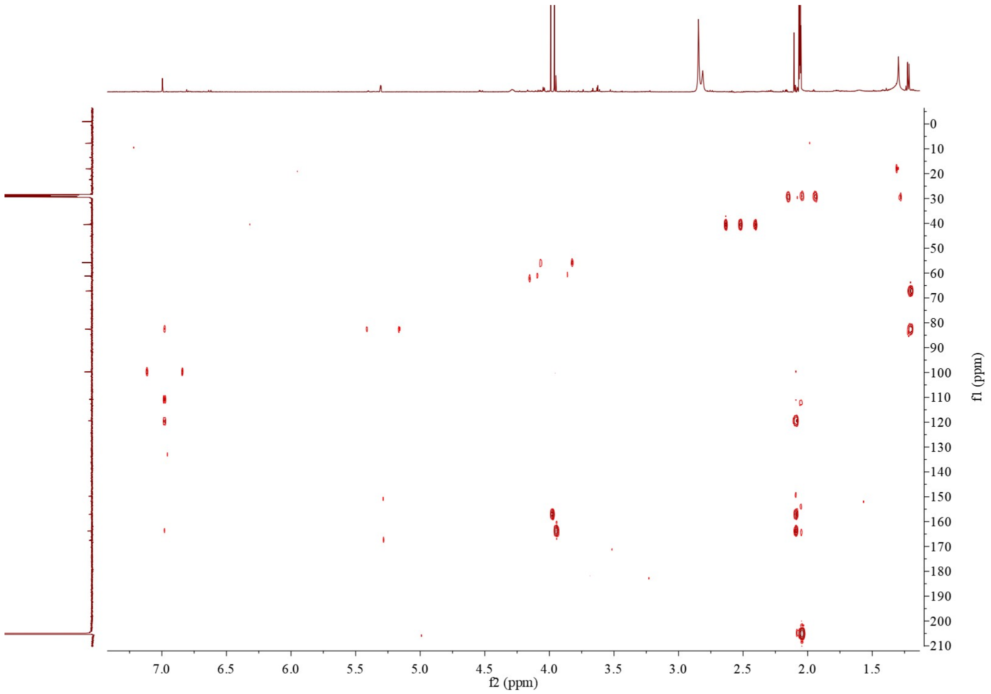

Figure S22 HMBC spectrum (600/150 MHz) of compound **4** in $(CD_3)_2CO$

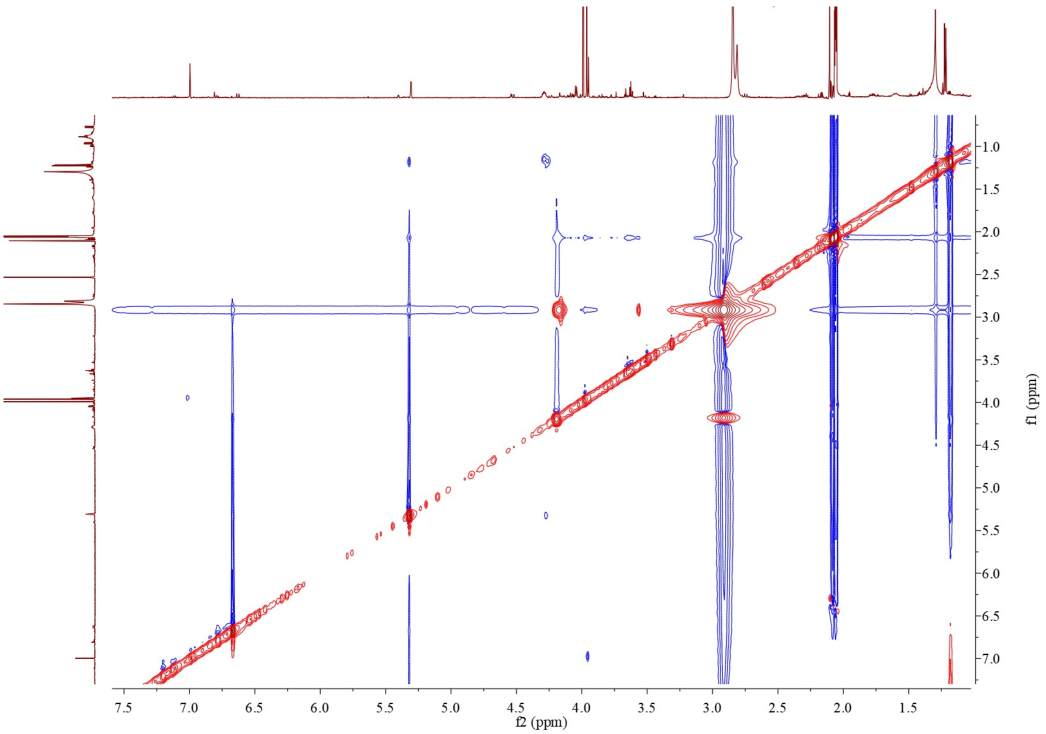

Figure S23 NOESY spectrum (600/150 MHz) of compound **4** in $(CD_3)_2CO$

Mass Spectrum SmartFormula Report

Analysis Info

Analysis Name	D:\Data\MS\data\201812\wuzhihui_QY00_pos_33_01_5897.d	Acquisition Date	12/09/2018 9:23:19 AM	
Method	LC_Direct Infusion_pos_70-500mz.m	Operator	SCSIO	
Sample Name	wuzhihui_QY00_pos	Instrument	maXis	255552.00029
Comment				

Acquisition Parameter

Source Type	ESI	Ion Polarity	Positive	Set Nebulizer	0.4 Bar
Focus	Active	Set Capillary	4500 V	Set Dry Heater	180 °C
Scan Begin	70 m/z	Set End Plate Offset	-500 V	Set Dry Gas	4.0 l/min
Scan End	1500 m/z	Set Charging Voltage	0 V	Set Divert Valve	Waste
		Set Corona	0 nA	Set APCI Heater	0 °C

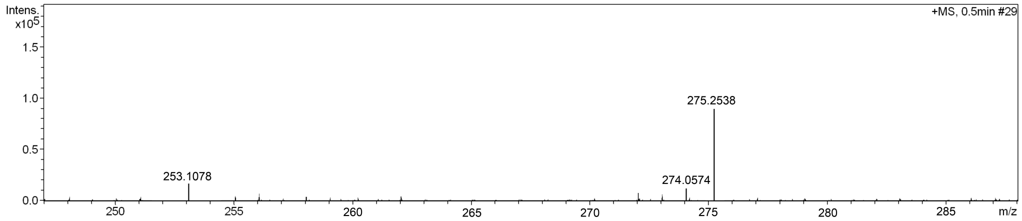

Meas. m/z	#	Ion Formula	Score	m/z	err [ppm]	err [mDa]	mSigma	rdb	e¯ Conf	N-Rule
253.1078	1	C13H17O5	100.00	253.1076	-0.4	-0.2	8.8	5.5	even	ok
275.2538	1	C13H16NaO5	100.00	275.2534	-1.5	-0.4	11.1	5.5	even	ok

Figure S24 HRESIMS spectrum of compound **4**

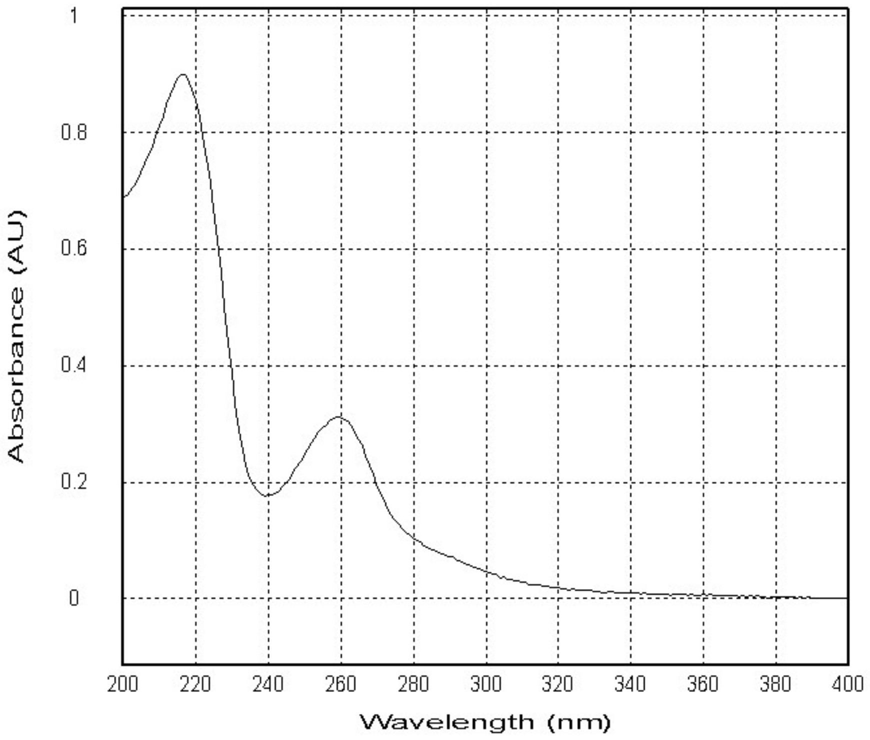

Figure S25 UV spectrum of compound **4**

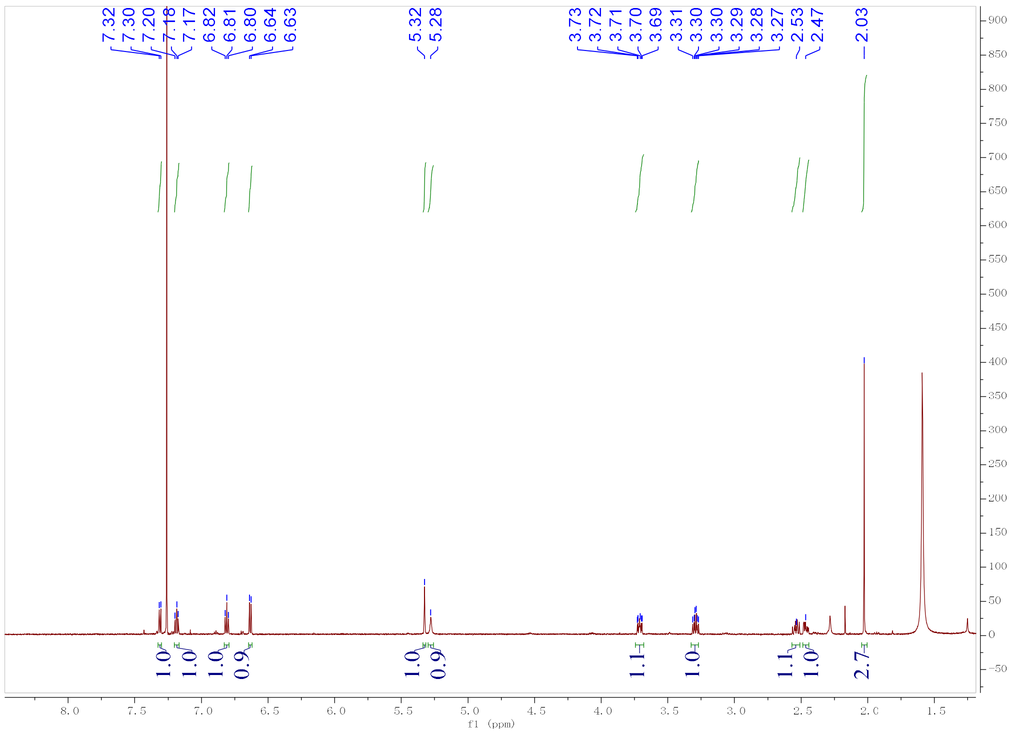

Figure S26 ^{1}H NMR spectrum (600 MHz) of compound **5** in $CDCl_3$

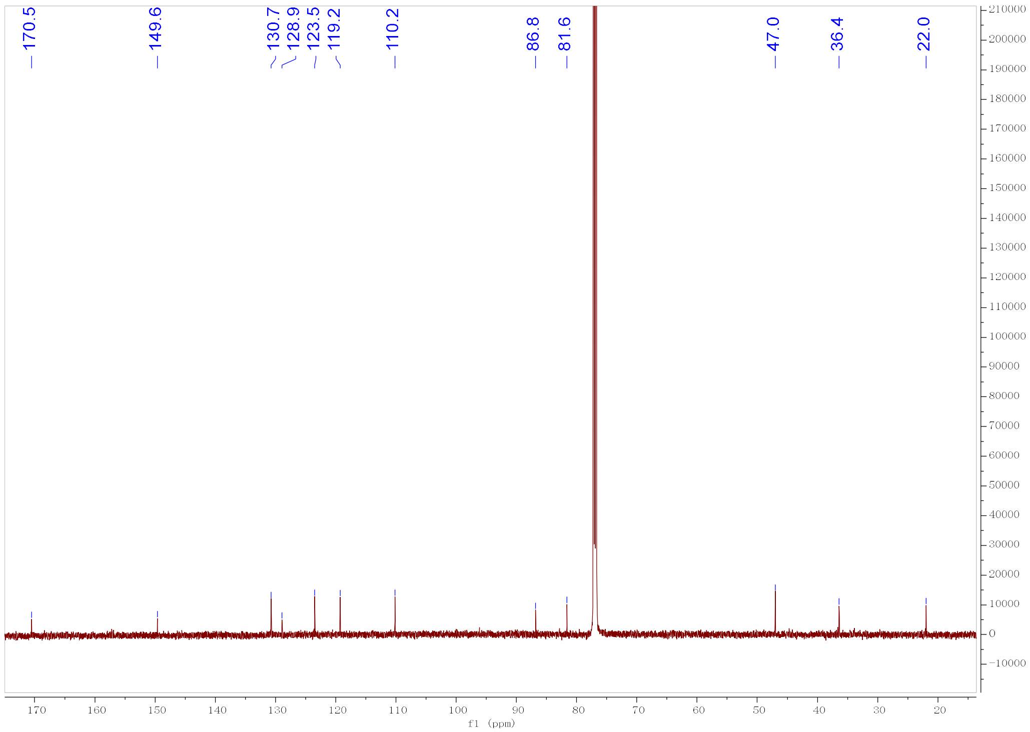

Figure S27 ^{13}C NMR spectrum (150 MHz) of compound **5** in $CDCl_3$

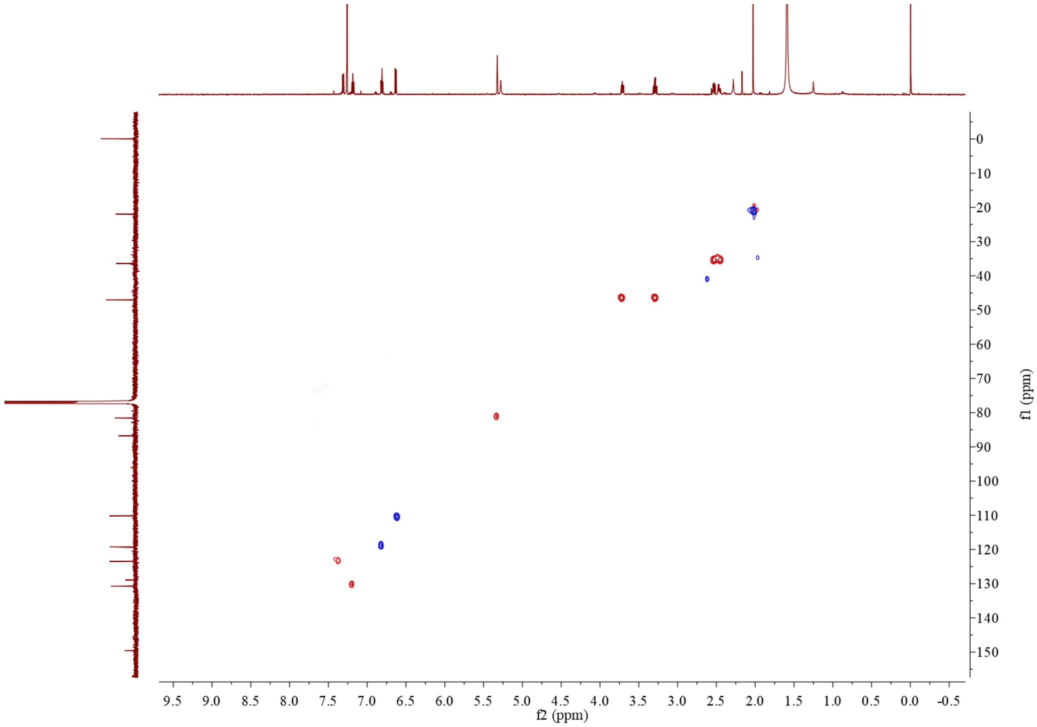

Figure S28 HMBC spectrum (600/150 MHz) of compound **5** in $CDCl_3$

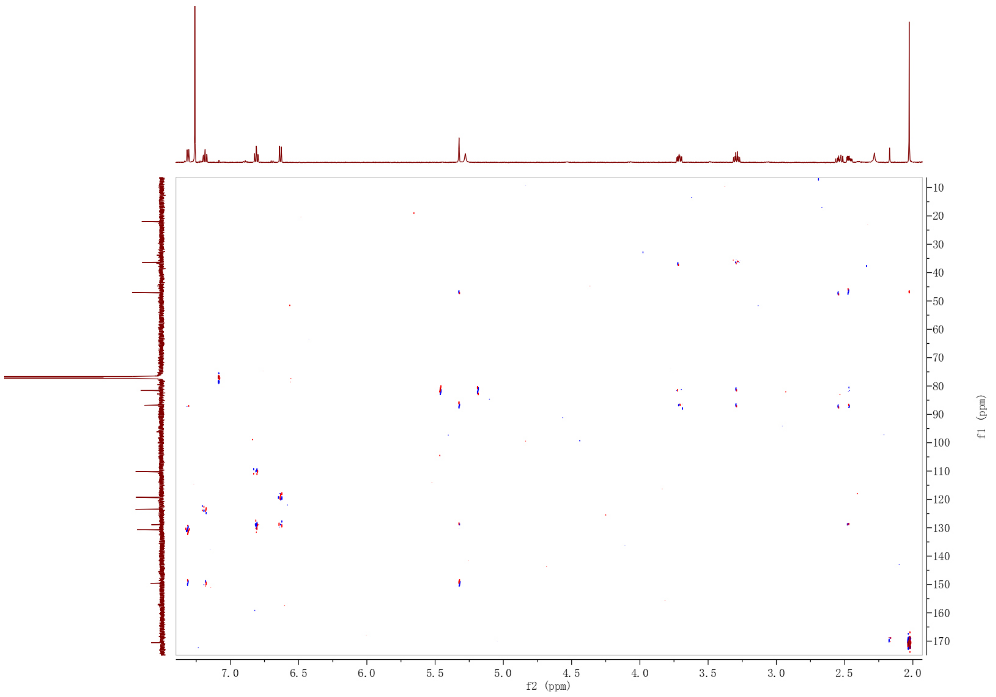

Figure S29 HMBC spectrum (600/150 MHz) of compound **5** in $CDCl_3$

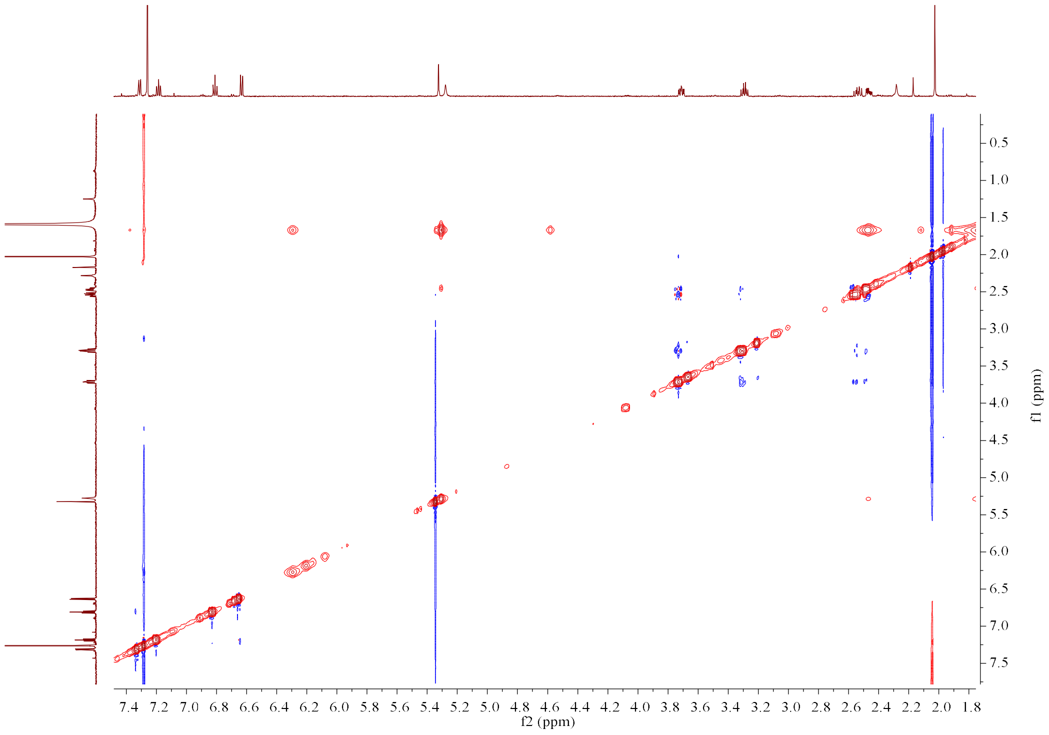

Figure S30 NOESY spectrum (600/150 MHz) of compound **5** in $CDCl_3$

Mass Spectrum SmartFormula Report

Analysis Info

Analysis Name	D:\Data\MS\data\201805\wuzhihui_hb67_pos_41_01_4818.d	Acquisition Date	5/24/2018 9:51:40 AM
Method	LC_Direct Infusion_pos_70-500mz.m	Operator	SCSIO
Sample Name	wuzhihui_hb67_pos	Instrument	maXis 255552.00029
Comment			

Acquisition Parameter

Source Type	ESI	Ion Polarity	Positive	Set Nebulizer	0.4 Bar
Focus	Active	Set Capillary	4500 V	Set Dry Heater	180 °C
Scan Begin	70 m/z	Set End Plate Offset	-500 V	Set Dry Gas	4.0 l/min
Scan End	1500 m/z	Set Charging Voltage	0 V	Set Divert Valve	Waste
		Set Corona	0 nA	Set APCI Heater	0 °C

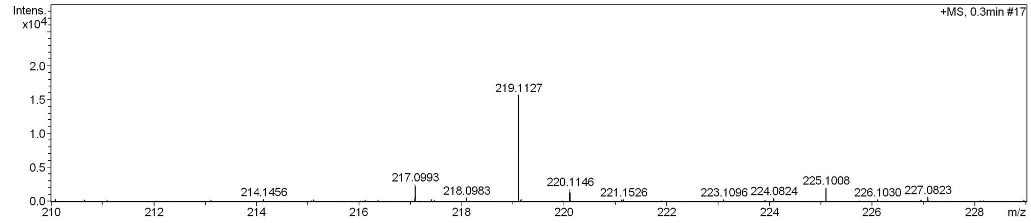

Meas. m/z	#	Ion Formula	Score	m/z	err [ppm]	err [mDa]	mSigma	rdb	e⁻ Conf	N-Rule
219.1127	1	C12H15N2O2	100.00	219.1128	0.5	0.1	13.1	6.5	even	ok

Figure S31 HRESIMS spectrum of compound **5**

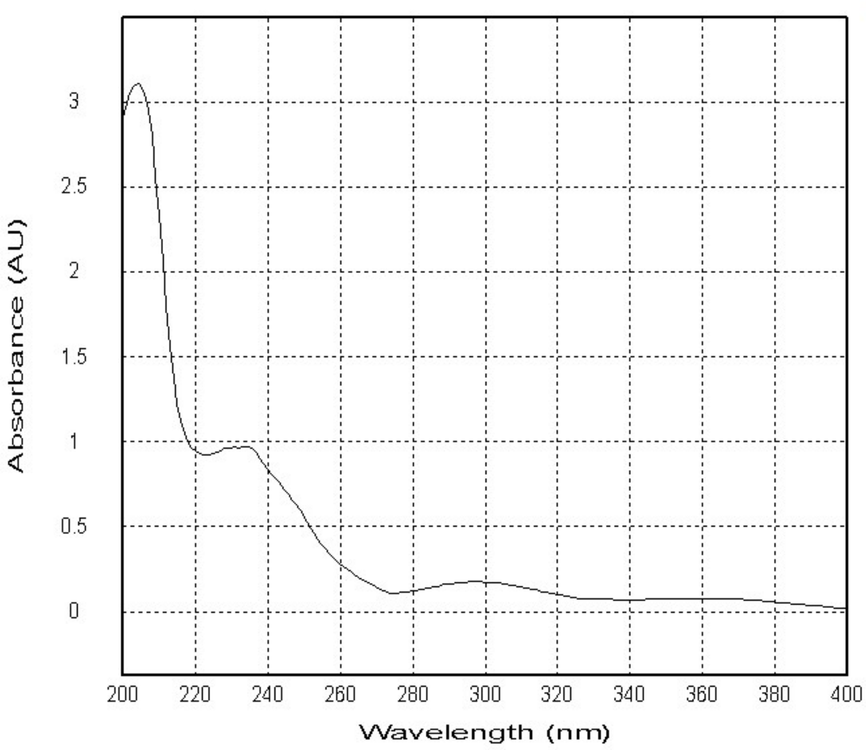

Figure S32 UV spectrum of compound **5**

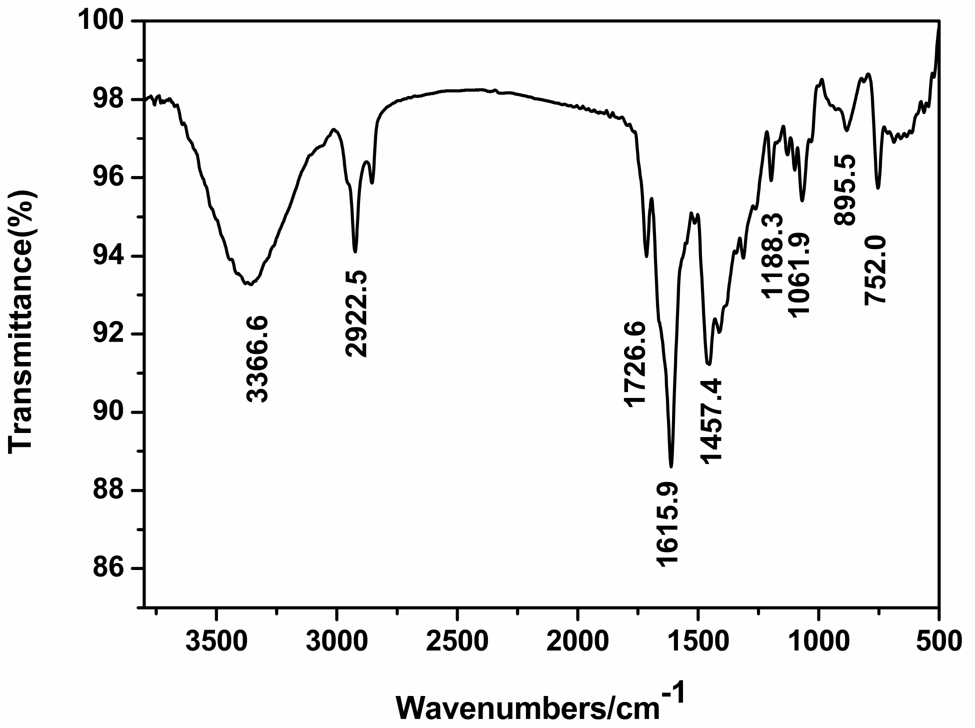

Figure S33 IR spectrum of compound **5**

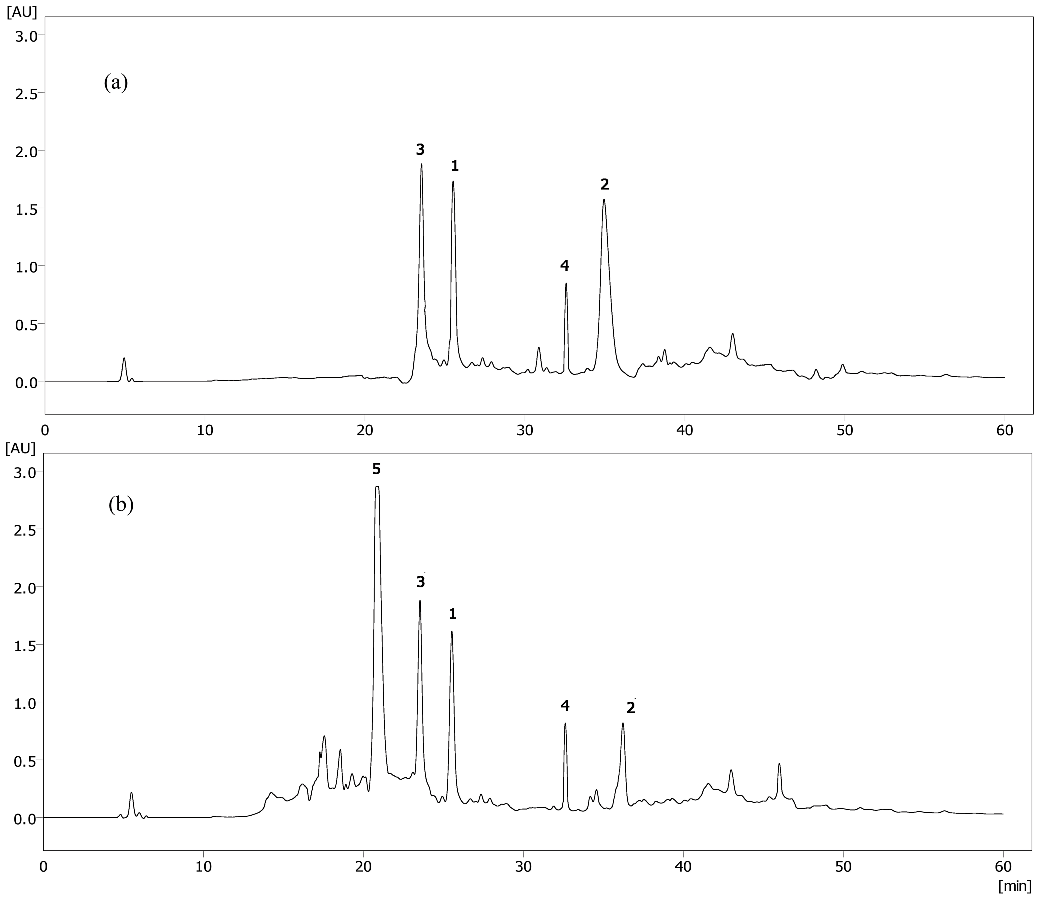

Figure S34 (a) HPLC profile of *B. ramosa* L29 EtOAc extract cultured in autoclaved rice medium; (b) HPLC profile of *B. ramosa* L29 EtOAc extract cultured in autoclaved rice medium with 0.25 mM (2*R*, 3*R*)-3, 5, 7-trihydroxyflavanone 3-acetate from *M. bontioides*. HPLC chromatograms (Hypersil BDS C18 column, 150 × 4.6 mm, 5 μm) using a gradient of MeOH/H_2O (20:80—80:20, 0–30 min; 80:20—100: 0, 30–45 min; 100: 0, 45–60 min) at a flow rate of 1.0 mL/min, and recorded at 254 nm).
